# Towards a Standardized Procedure for the Production of Infective Spores to Study the Pathogenesis of Dermatophytosis

**DOI:** 10.3390/jof7121029

**Published:** 2021-11-30

**Authors:** Emilie Faway, Cindy Staerck, Célya Danzelle, Sophie Vroomen, Christel Courtain, Bernard Mignon, Yves Poumay

**Affiliations:** 1 Molecular Physiology Research Unit, NAmur Research Institute for Life Sciences (URPHYM-NARILIS), Faculty of Medicine, University of Namur, 5000 Namur, Belgium; emilie.faway@unamur.be (E.F.); christel.courtain@student.unamur.be (C.C.); 2 Fundamental and Applied Research for Animals & Health (FARAH), Faculty of Veterinary Medicine, University of Liège, 4000 Liège, Belgium; cindy.staerck@uliege.be (C.S.); celya.danzelle@uliege.be (C.D.); sophie.vroomen@uliege.be (S.V.)

**Keywords:** dermatophytosis, dermatophytes, infective spores, arthroconidia, microconidia, reconstructed human epidermis, dermatophyte pathogenesis, experimental models, *Trichophyton*, *Microsporum*

## Abstract

Dermatophytoses are superficial infections of human and animal keratinized tissues caused by filamentous fungi named dermatophytes. Because of a high and increasing incidence, as well as the emergence of antifungal resistance, a better understanding of mechanisms involved in adhesion and invasion by dermatophytes is required for the further development of new therapeutic strategies. In the last years, several in vitro and in vivo models have emerged to study dermatophytosis pathogenesis. However, the procedures used for the growth of fungi are quite different, leading to a highly variable composition of inoculum for these models (microconidia, arthroconidia, hyphae), thus rendering difficult the global interpretation of observations. We hereby optimized growth conditions, including medium, temperature, atmosphere, and duration of culture, to improve the sporulation and viability and to favour the production of arthroconidia of several dermatophyte species, including *Trichophyton rubrum* and *Trichophyton benhamiae*. The resulting suspensions were then used as inoculum to infect reconstructed human epidermis in order to validate their ability to adhere to and to invade host tissues. By this way, this paper provides recommendations for dermatophytes culture and paves the way towards a standardized procedure for the production of infective spores usable in in vitro and in vivo experimental models.

## 1. Introduction

Dermatophytoses are superficial infections of the skin, hair, and nails due to filamentous and keratinolytic fungi named dermatophytes [1]. Currently, dermatophytoses constitute the most common mycosis worldwide, affecting 20–25% of the global population [2], and up to 50% among populations at risk, such as sportsmen and diabetic patients [3,4,5]. Genetic predispositions also exist and heredity participates, along with lifestyle, in the increase of dermatophytosis prevalence observed in the last decades [6,7]. Classical antifungals, such as azole derivatives and terbinafine, are generally efficient to treat dermatophytosis [8], but their use is associated with potential toxicity when systemic administration is required [9] and must cope with the growing emergence of resistant strains [10,11,12,13,14].

Because of this high and still increasing prevalence of dermatophytosis, as well as the difficulties encountered with currently available treatments, more research is needed to elucidate mechanisms responsible for fungal adherence and invasion of the host tissue. For this purpose, several in vitro and in vivo models of infection by dermatophytes have been developed (for reviews, see [15,16] respectively). 

Experimental infection models described in the literature use different compositions of inoculum, consisting in suspensions enriched in unicellular infective spores, namely microconidia [17,18,19,20,21,22,23] or arthroconidia [24,25,26,27,28,29], or in a mix of microconidia and hyphae [30,31,32,33,34,35]. Microconidia, as well as macroconidia, are a type of thallic spores resulting from lateral or terminal budding of hyphae. Although micro- and macroconidia are easier to produce in culture, and thus frequently used as infective spores in most experimental models, they have never been observed in natural lesions [36]. In contrast, arthroconidia, produced by fragmentation and disarticulation of hyphae septate [37,38], are recognized as natural vectors of dermatophytosis [36]. However, even though their use appears more representative of natural infection, arthroconidia are less frequently used in experimental models since their formation is particularly difficult to induce in vitro. Arthroconidia production was nevertheless documented in some studies using *T. mentagrophytes* [37,39,40,41,42] or *M. canis* [25], and more recently with *T. rubrum* [43,44]. 

This heterogeneity in the composition of inoculum, produced in various conditions of culture, including media, temperature, atmosphere, or duration, could influence the development of infection, rendering impossible any comparison between the observations obtained from different experimental models. Thus, overall conclusions about the pathogenic mechanisms responsible for dermatophytosis remain hard to define. In this paper, we assessed the growth and production of spores, especially arthroconidia, by several dermatophyte species frequently isolated from human lesions, in different culture conditions, with the aim to define a standardized procedure for the production of infective spores usable as inoculum in experimental in vitro and in vivo models. In addition, the infectious potential of these inocula was checked through an evaluation of infection development in a model of reconstructed human epidermis (RHE).

## 2. Materials and Methods

### 2.1. Dermatophyte Strains

This study was conducted on a panel of 13 dermatophyte strains described in Table 1 and obtained from IHEM (Institute of Hygiene and Epidemiology-Mycology section) from the Scientific Institute of Public Health (Brussels, Belgium). The strains were firstly grown on a homemade Sabouraud (SAB) medium (Table 2) plate at 30 °C for 14 days.

### 2.2. Growth Evaluation

The growth capacity of dermatophytes was determined using five different agar media: Sabouraud (SAB), potato dextrose agar (PDA), yeast peptone dextrose (YPD), malt medium (MALT), and yeast extract nitrogen (YEN). The composition of each medium, as well as the suppliers for all ingredients used, are described in Table 2. Practically, fungi were grown on SAB at 30 °C for 14 days, after which the fungal material was scraped with a scalpel blade and transferred in PBS (pH 7.2). Fungal suspensions were centrifuged at 3000× *g* for 5 min, before pellets were washed three times with PBS and finally resuspended in an adequate volume of PBS to obtain a concentration of 1 × 10^5^ colony-forming unit (CFU) per mL. Five microliters of the resulting fungal suspension were then spotted on the centre of each medium plate and incubated in the dark at 30 °C. The diameter of fungal colonies was measured every day for 31 days and radial growths were determined during the exponential phase by analysis of the growth curves. 

### 2.3. Sporulation Evaluation

The sporulation of dermatophytes was assessed after 31 days of growth at 30 °C on the five used agar media (SAB, PDA, YPD, MALT and YEN, Table 2). Practically, the fungal material was scrapped from the surface of the agar plate with a scalpel blade and suspended in PBS (pH 7.2). Recovered suspensions were then filtered through three Miracloth layers (22–25 µm pore size; cat. no. 475855; Merck KGaA, Darmstadt, Germany) to remove hyphae and centrifuged at 3000× *g* for 5 min. Pellets were washed three times with PBS and resuspended in 5 mL PBS. Total spore concentration, including microconidia, macroconidia, and arthroconidia, was microscopically enumerated using a Thoma cell counting chamber. 

### 2.4. Production of Infective Spore Suspensions 

The production of a suspension of infective spores adequate for use in experimental models was evaluated on three different homemade media (SAB, PDA and YEN, Table 2). Briefly, after growth on SAB for 14 days, the fungal material was scraped with a scalpel blade, transferred in PBS (pH 7.2), and seeded on SAB, PDA, or YEN media for sporulation. The plates were incubated at 30 °C under 12% CO_2_ or at 37 °C under 10% CO_2_, for 10, 21, or 31 days. Spore suspensions were then recovered as previously described in Section 2.3, with an additional step of stirring in order to efficiently dissociate arthroconidia and microconidia from hyphae. 

The viable fungal concentration of the resulting suspensions was determined by CFU counting of serial dilutions after three days of incubation at 30 °C on SAB, and the total spore concentration was determined under light microscopy as described in Section 2.3. Simultaneously, the proportion of arthroconidia was calculated relative to the total spore concentration after examining five randomly selected microscopical fields under ×400 magnification. The spore suspensions were finally observed and pictured under differential interference contrast (DIC) microscopy (Olympus; Tokyo, Japan).

### 2.5. Infection Development on Reconstructed Human Epidermis

Initially, the inoculum used in our model of infection on RHE consisted in a suspension of spores recovered after a 21-days incubation on YEN at 30 °C under 12% CO_2_ [28]. Based on our previous results, herein, we compared inoculum prepared using this conventional procedure with a spore suspension recovered after a 10-days incubation on PDA at 30 °C under 12% CO_2_, appearing as the optimal method to obtain a high viable fungal concentration. The preparation of these spore suspensions was performed as described in Section 2.4. Spores were stored at 4 °C and used within one month. 

The assays of infection on RHE were performed for *T. benhamiae* IHEM 20163 and *T. rubrum* IHEM 13894 strains. The inoculum was adjusted at the required quantity by dilution in PBS: 30 CFU for *T. benhamiae* and 1000 CFU for *T. rubrum*, respectively corresponding to densities of about 53 per cm^2^ and 1700 per cm^2^.

#### 2.5.1. Production of RHE and Infection by Dermatophytes

RHE were prepared as previously described [46]. Briefly, normal human keratinocytes were isolated from skin samples obtained from plastic surgery (Dr. Bienfait and Dr. Blairvacq, Clinique St-Luc, Namur-Bouge, Belgium). After prior amplification in EpiLife medium (cat. no. M-EPI-500-CA; Life Technologies, Waltham, MA, USA) supplemented with Human Keratinocyte Growth Supplement (HKGS; cat. no. S-001-5; Life Technologies, Waltham, MA, USA), primary keratinocytes were seeded at a high density of 250,000 cells per cm^2^ onto polycarbonate culture insert characterized by pores of 0.4 µm diameter (cat. no. PIHP01250; Merck KGaA, Darmstadt, Germany) in EpiLife medium supplemented with HKGS and 1.5 mM Ca^2+^. After 24 h, keratinocytes were exposed to the air-liquid interface by removing the culture medium above the insert, while the medium under the insert was replaced by EpiLife medium supplemented with HKGS, 1.5 mM Ca^2+^, 10 ng/µL keratinocytes growth factor (KGF; cat. no. 251-KG; R&D Systems, Abingdon, UK) and 50 µg/mL vitamin C (2-phospho-L-ascorbic acid trisodium salt; cat. no. 49752; Merck KGaA, Darmstadt, Germany). Keratinocytes were maintained at 37 °C in a humidified atmosphere containing 5% CO_2_ with medium under the insert renewed every two days. After 11 days, fully differentiated epidermis can be obtained and used for infection by dermatophytes. 

Infection of RHE by dermatophytes was performed as described in Faway et al. [28]. Practically, an inoculum made of infective spore suspensions, produced as depicted in Section 2.4, was topically applied onto the surface of RHE on the day of infection. At this step, an additional inoculum was seeded onto SAB plates to monitor the fungal load by counting CFU after three days of incubation at 30 °C (internal control). Four hours after infection, the surface of RHE was rinsed three times with PBS in order to eliminate non-adherent spores and to expose again the cultured keratinocytes to the air–liquid interface. Non-adherent spores recovered during washes were counted onto SAB, in order to evaluate the fraction of remaining adherent spores. Finally, infected RHE were maintained in culture for four additional days with culture medium and the tissue was renewed every day.

#### 2.5.2. Histological Analysis

Infected RHE were recovered each day during the four days following infection, fixed in 4% formaldehyde solution, and histologically processed before periodic-acid Schiff (PAS) staining with pre-treatment using α-amylase and hemalun counterstaining, as described by Faway et al. [28].

#### 2.5.3. Measurement of Infection Level

The quantification of the infection level on RHE was performed by the assessment of the copy number of the dermatophyte 18S rDNA gene using qPCR as previously described [28]. Briefly, total DNA was extracted from infected RHE using the DNeasy^®^ Blood & Tissue Kit (cat. no. 69504; Qiagen, Hilden, Germany) after tissue homogenization using Tissue Grinder (cat. no. NG010; NIPPON Genetics EUROPE, Duren, Germany). Quantitative PCR was then performed using Light Cycler 96 (Roche, Basel, Switzerland) and the PCR mixture was made of Takyon^TM^ No ROX SYBR^®^ Master Mix (cat. no. UF-NSMT-B0701; Eurogentec, Seraing, Belgium), 300 nM of each primer, and 20 ng of DNA. Primer sequences were chosen to amplify the 18S rDNA gene from several dermatophyte species, including *T. rubrum* and *T. benhamiae* (18SrDNA-F 5′-TAACGAACGAGACCTTAACC-3′ and 18SrDNA-R 5′-TTATTGCCTCAAACTTCCAT-3′, described in Paugam et al. [47]). Absolute quantification was realized thanks to a standard curve made of known 18S rDNA copy number samples. These samples were obtained after DNA extraction from *T. rubrum* or *T. benhamiae* mycelium grown on SAB plates, PCR amplification of the 18S rDNA gene using the Platinum^TM^ SuperFi^TM^ DNA Polymerase (cat. no. MAN0014882; Invitrogen, Carlsbad, CA, USA), and purification by the MinElute^®^ PCR Purification Kit (cat. no. 28004; Qiagen, Hilden, Germany). For more details, please refer to Faway et al. [28].

#### 2.5.4. Epidermal Barrier Integrity Monitoring

The integrity of the RHE epidermal barrier was monitored during infection by measurement of the trans-epithelial electrical resistance (TEER) using a Millicell ERS-2 Voltohmmeter (cat. no. MERS00002; Merck KGaA, Darmstadt, Germany). These measurements are expressed as a percent of TEER values measured through RHE before infection. 

#### 2.5.5. RNA Extraction, Reverse Transcription and Quantitative PCR

Expression of proinflammatory cytokines and antimicrobial peptides by keratinocytes during infection of RHE was assessed by RT-qPCR after total RNA extraction. RPLP0 was used as a reference gene [48] and results are expressed as relative expression compared to control non-infected RHE. Practically, total RNA was extracted from infected and control RHE using RNeasy Mini Kit (cat. no. 74104; Qiagen, Hilden, Germany) according to the manufacturer’s instructions, and 1 µg of RNA was reverse-transcribed into cDNA using the SuperScript III Reverse Transcriptase kit (cat. no. 18080; Invitrogen, Carlsbad, CA, USA). The qPCR mixture contained Takyon^TM^ No ROX SYBR^®^ Master Mix (cat. no. UF-NSMT-B0701; Eurogentec, Seraing, Belgium), 300 nM of each primer and 5 µL of cDNA diluted 1:20 in distilled water. The qPCR was performed using Light Cycler 96 (Roche, Basel, Switzerland). All primers used for qPCR were designed in-house using Primer-BLAST online software (https://www.ncbi.nlm.nih.gov/tools/primer-blast/ accessed on 1 September 2021) and showed efficacy between 90% and 110% [29]. Primers sequences are listed in Table 3. 

#### 2.5.6. ELISA Assays

The release of the proinflammatory cytokines IL-1α and IL-8 by keratinocytes during infection of RHE was monitored by ELISA assay performed on culture medium. Commercial ELISA kits, Human IL-1 alpha/IL1F1 DuoSet ELISA and Human IL-8/CXCL8 DuoSet ELISA (cat. no. DY200 and DY208; R&D Systems, Abingdon, UK), were used according to the manufacturer’s instructions. 

### 2.6. Statistical Analysis

Results for each assay are expressed as the means with standard deviations, or individual values, of at least triplicate experiments performed independently and using freshly prepared fungal suspensions. Data were plotted using GraphPad Prism 9 software. Results were analysed by one-way (ANOVA1) or two-way (ANOVA2) variance analysis using GraphPad Prism 9 and SigmaPlot version 11.0 software, and were considered as significantly different with a *p*-value < 0.05.

## 3. Results

### 3.1. Growth and Sporulation of Dermatophytes Are Improved on PDA

**Figure 1:**
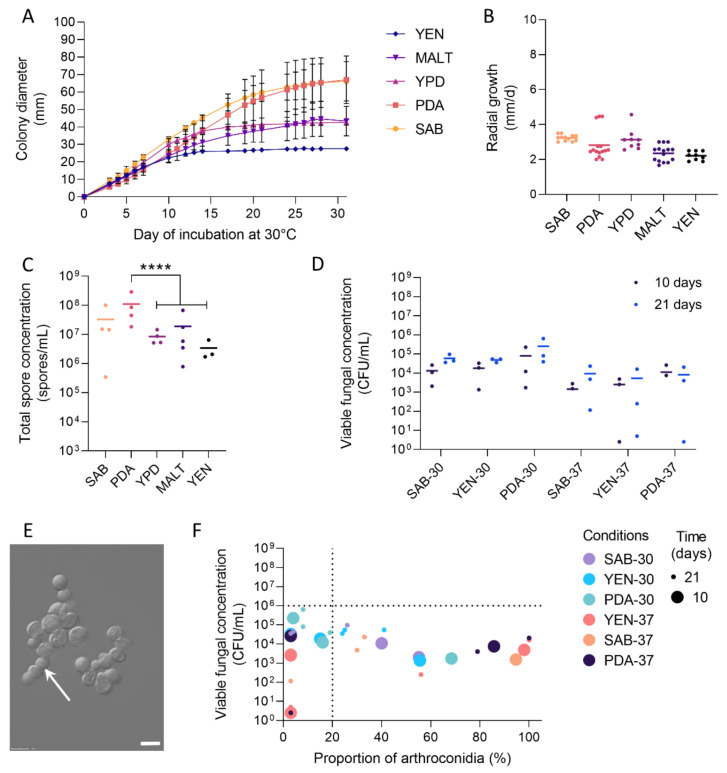
Growth and sporulation of *T. rubrum* IHEM 13894. *T. rubrum* IHEM 13894 were seeded over five agar media, Sabouraud (SAB), Potato Dextrose Agar (PDA), Yeast Peptone Dextrose (YPD), Malt medium (MALT) or Yeast Extract Nitrogen (YEN), and grown at 30 °C for 31 days. Fungal material was then scrapped, suspended in PBS and filtered to obtain fungal suspensions. (**A**) Diameter of fungal colonies was measured daily, (**B**) radial growth was determined during the exponential phase of growth, and (**C**) the total spore concentration recovered in fungal suspension was determined by counting using a Thoma chamber under light microscopy. To promote sporulation and arthroconidial production by *T. rubrum* IHEM 13894, fungi were seeded on SAB, PDA or YEN media, and incubated at 30 °C under 12% CO_2_ or at 37 °C under 10% CO_2_, for 10 or 21 days. Fungal material was then scrapped, suspended in PBS, stirred and filtered to obtain spore suspensions. (**D**) Viable fungal concentration was determined by counting colony-forming units (CFU). (**E**) Morphology of arthroconidia (white arrow) was observed using differential interference contrast (DIC) microscopy. Scale bar = 10 µm. (**F**) Viable fungal concentration was correlated with the proportion of arthroconidia relative to the total number of fungal elements, determined by counting using a Thoma chamber under light microscopy. Dotted lines indicate the minimal viable fungal concentration required for further use on experimental models, based on the most important published models [15,16] and personal observations, as well as the proportion of 20% arthroconidia, arbitrarily chosen as a minimal value for suspensions enriched in arthroconidia. Data analysis: n ≥ 3; means ± SD for (**A**), means and individual values for (**B**–**D**), and single plots for (**F**); ANOVA2 for (**B**–**D**) compared to PDA; **** *p* < 0.0001.

**Figure 2:**
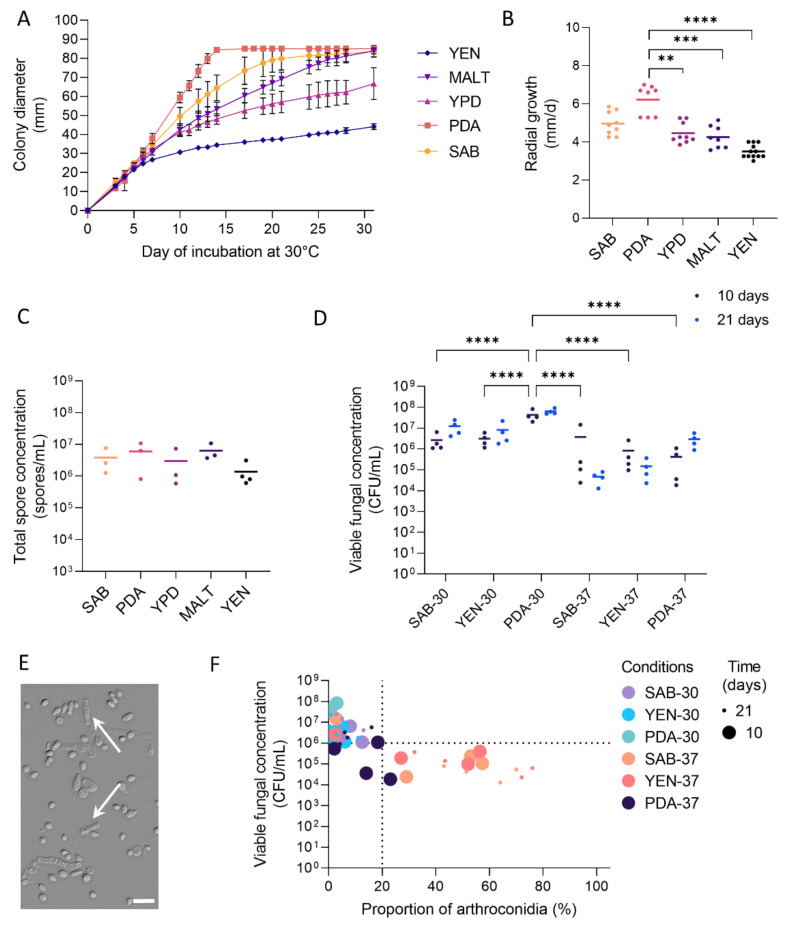
Growth and sporulation of *T. benhamiae* IHEM 20163. *T. benhamiae* IHEM 20163 were seeded over five agar media, Sabouraud (SAB), Potato Dextrose Agar (PDA), Yeast Peptone Dextrose (YPD), Malt medium (MALT) or Yeast Extract Nitrogen (YEN), and grown at 30 °C for 31 days. Fungal material was then scrapped, suspended in PBS and filtered to obtain fungal suspensions. (**A**) Diameter of fungal colonies was measured daily, (**B**) radial growth was determined during the exponential phase of growth, and (**C**) the total spore concentration recovered in fungal suspension was determined by counting using a Thoma chamber under light microscopy. To promote sporulation and arthroconidial production by *T. benhamiae* IHEM 20163, fungi were seeded on SAB, PDA or YEN media, and incubated at 30 °C under 12% CO_2_ or at 37 °C under 10% CO_2_, for 10 or 21 days. Fungal material was then scrapped, suspended in PBS, stirred and filtered to obtain spore suspensions. (**D**) Viable fungal concentration was determined by counting colony-forming units (CFU). (**E**) Morphology of arthroconidia (white arrow) was observed using differential interference contrast (DIC) microscopy. Scale bar = 10 µm. (**F**) Viable fungal concentration was correlated with the proportion of arthroconidia relative to the total number of fungal elements, determined by counting using a Thoma chamber under light microscopy. Dotted lines indicate the minimal viable fungal concentration required for further use on experimental models, based on the most important published models [15,16] and personal observations, as well as the proportion of 20% arthroconidia, arbitrarily chosen as a minimal value for suspensions enriched in arthroconidia. Data analysis: n ≥ 3; means ± SD for (**A**), means and individual values for (**B**–**D**), and single plots for (**F**); ANOVA2 for (**B**–**D**) compared to PDA; ** *p* < 0.01 *** *p* < 0.001 **** *p* < 0.0001.

The growth of dermatophytes was monitored on different media (SAB, PDA, YPD, MALT, and YEN) by determination of colony diameter and radial growth. Strangely, YEN medium, classically used for the production of spore suspensions of *M. canis* appeared not suitable for *Trichophyton* species (Figure S1B). Globally, the colony diameters of all the strains used in this study were higher on PDA than on the other media (Figure S1A) and these data were in accordance with the radial growth determined on PDA (Figure S2). Notably, *T. rubrum* IHEM 13894 presented a diameter about twofold superior on SAB and PDA as compared to other media, without significant improvement of the radial growth (Figure 1A,B and Figure S3). For *T. benhamiae* IHEM 20163, the colony size after 10 days was even more enhanced on PDA than on SAB (Figure 2A,B and Figure S3). In addition, all the strains, except *M. audouinii* IHEM 10316, *T. rubrum* IHEM 13894, and *T. tonsurans* IHEM 24958, exhibited a reproductive fungal growth curve on PDA, with few variations between replicate assays.

In parallel to growth, the sporulation of dermatophytes was assessed on each tested medium by determining the total spore concentration after 31 days of incubation, when the strains were in stationary phase on most media. Spores were mainly represented by microconidia and to a lesser extent by arthroconidia, with macroconidia being extremely rare due to the porosity of the Miracloth filter used for spore production (see Section 2.3 in Section 2). On PDA, the amount of spores produced by *T. rubrum* IHEM 13894 was significantly higher than on YPD, MALT, and YEN (Figure 1C). The same trend, whereas not statistically significant, was observed for *T. benhamiae* IHEM 20163 (Figure 2C). Consistently, PDA significantly increased the total spore concentration for *M. canis* IHEM 22957 compared to SAB, YPD, and YEN, and for *T. mentagrophytes* IHEM 25841 and TIMM 2789 compared to YEN (Figure S4). For all other strains, sporulation was higher on PDA than on the other media, but without significant difference. 

### 3.2. High Viable Fungal Concentration Is Obtained after Incubation on PDA 

In order to produce fungal suspensions suitable for use in experimental models, i.e., containing mainly unicellular spores (microconidia and ideally arthroconidia) with sufficient concentration and viability (>10^6^ CFU/mL), dermatophytes were incubated for 10 or 21 days on SAB, YEN, and PDA at 30 °C under 12% CO_2_ or at 37 °C under 10% CO_2_. After the recovery of spore suspensions, the viable fungal concentration was evaluated by CFU counting. 

For all the strains, except *T. tonsurans* IHEM 24958 and *M. audouinii* IHEM 10316, growth on PDA at 30 °C under 12% CO_2_ resulted in sufficient quantity of viable fungal elements (Figure 1D, Figure 2D and Figures S4–S6). Furthermore, for *Trichophyton* species, an incubation period of 10 days rather than 21 days did not have a significant negative impact on viable fungal concentration. For some strains, the variability of the fungal concentration obtained between replicates was even reduced, ultimately allowing a reduction in the culture time required. In contrast, for *Microsporum* species, a culture duration of less than 21 days appeared inappropriate due to lower viable fungal concentration.

For *M. audouinii* IHEM 10316 and *T. tonsurans* IHEM 24958, viable fungal concentration was extremely low, i.e., less than 10^4^ CFU/mL, irrespective of conditions and media (Figures S5 and S7). Extending the incubation time to 31 days somehow increased the viable concentration, but it remained low and insufficient for further use in experiments. 

### 3.3. Production of Arthroconidia Is Highly Variable According to Strains and Culture Conditions

Arthroconidia were observed using DIC microscopy and counted using a Thoma chamber under light microscopy to determine their percentage in the spore suspensions (see Section 2.4). In *Microsporum* species, the arthroconidia were rather rectangular in shape, with straight or curved edges, respectively for *M. audouinii* and *M. canis* (Figure S8). In contrast, their morphology was highly variable among *Trichophyton* species, with rectangular, spherical, or ovoid shapes (Figure 1E, Figure 2E and Figure S8). Furthermore, the morphology of arthroconidia may differ between their formation inside the hyphae and their release as individual cells, as for example arthroconidia of *T. interdigitale* appearing rectangular-shaped inside hyphae and spherical-shaped once they became individuals, while the contrary was observed for the arthroconidia of *T. mentagrophytes* (Figure S8). 

The percentage of arthroconidia in spore suspensions varied considerably between culture conditions and between replicates for a given condition (Figure 1F, Figure 2F, and Figure S9). Furthermore, for each of the dermatophyte strains, the conditions favouring arthroconidial production, and those allowing sufficient viability (>10^6^ CFU/mL) to be achieved, were not the same. Indeed, using a culture on PDA at 30 °C under 12% CO_2_ for 10 (*Trichophyton* spp.) or 21 days (*Microsporum* spp.), a condition defined as optimal in terms of viability, the proportion of arthroconidia was extremely variable and quite low (<20%) for the majority of strains (Table 4, Figure S10). 

### 3.4. Spore Suspensions Recovered after 10 Days on PDA at 30 °C under 12% CO_2_ Are Adequate to Induce Infection on RHE

Mainly based on the viable fungal concentration, a 10-day (21-days for *Microsporum* species) incubation on PDA at 30 °C under 12% CO_2_ appeared to be the optimal condition for the production of spore suspensions suitable as inoculum in experimental models. Indeed, in such conditions, the viable fungal concentration was above 10^6^ CFU/mL, a concentration sufficient to induce infection when used in in vitro [15] and in vivo models [16]. Nevertheless, no information is available in the literature on the pathogenicity of spore suspensions of dermatophytes produced in such conditions. Thus, the infectious ability of these suspensions was evaluated in RHE model. Since the dermatophytosis model on RHE was initially developed using spore suspensions recovered after 21 days of incubation on YEN at 30 °C under 12% CO_2_ as an inoculum [28], herein, we compared the infection development on RHE after topical application of spore suspensions recovered following the “conventional procedure” (21 days on YEN) or the “optimized procedure” (10 days on PDA). This comparison of the infection development was performed for the strains *T. rubrum* IHEM 13894 and *T. benhamiae* IHEM 20163. The size of inoculum was determined to induce a fungal invasion restricted to the cornified layer, as can be observed during natural in vivo infection, and appeared to be 1000 CFU for *T. rubrum* and 30 CFU for *T. benhamiae* [28]. 

#### 3.4.1. Adherence to RHE Is Improved for Spores Recovered on PDA 

**Figure 3:**
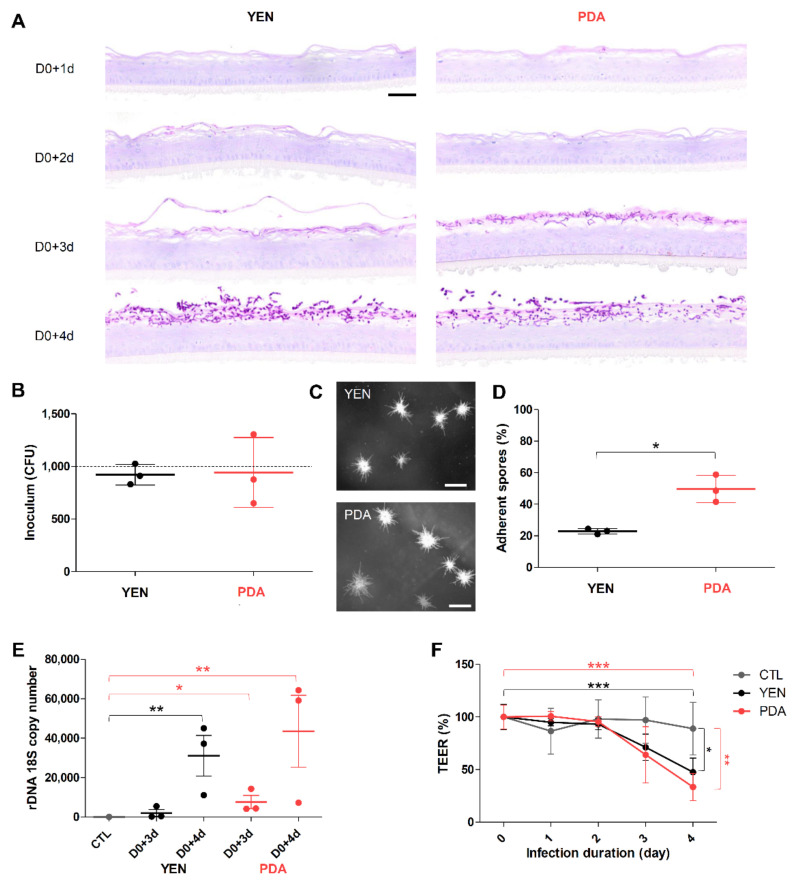
*T. rubrum* IHEM 13894 infection on reconstructed human epidermis (RHE). RHE were infected on day zero (D0) by topical addition of inoculum consisting in suspensions of *T. rubrum* IHEM 13894 spores recovered after 21 days of incubation over Yeast Extract Nitrogen agar (YEN) at 30 °C under 12% CO_2_, or after 10 days of incubation over Potato Dextrose Agar (PDA) in the same conditions. The inoculum was adjusted to a density of 1000 colony-forming units (CFU) per RHE. (**A**) Histological sections were prepared from infected RHE analysed one (D0+1d), two (D0+2d), three (D0+3d) or four (D0+4d) days after infection and then stained using the Periodic-acid Schiff (PAS) procedure with α-amylase pre-treatment and hemalun counterstaining. Scale bar = 50 µm. (**B**) Fungal load of inoculum was monitored by counting CFU, the dotted line indicates the expected size of inoculum, and (**C**) the macroscopic aspect of CFU was observed. Scale bars = 1 mm. (**D**) Percentage of spores adhering to the epidermis was determined four hours after infection. (**E**) Infection levels were assessed three (D0+3d) and four (D0+4d) days after infection of RHE using qPCR analysis of the rDNA 18S gene copy numbers of *T. rubrum* in total DNA extracts prepared from infected RHE (YEN and PDA) or non-infected RHE (CTL). (**F**) Epidermal barrier integrity of RHE during infection was determined by measurements of trans-epithelial electrical resistance (TEER). TEER values are expressed as percentage of values measured before RHE infection (D0). Data analysis: n = 3; means ± SD; ANOVA1 for (**B**,**D**), ANOVA2 for (**E**,**F**); * *p* < 0.05 ** *p* < 0.01 *** *p* < 0.001.

**Figure 4:**
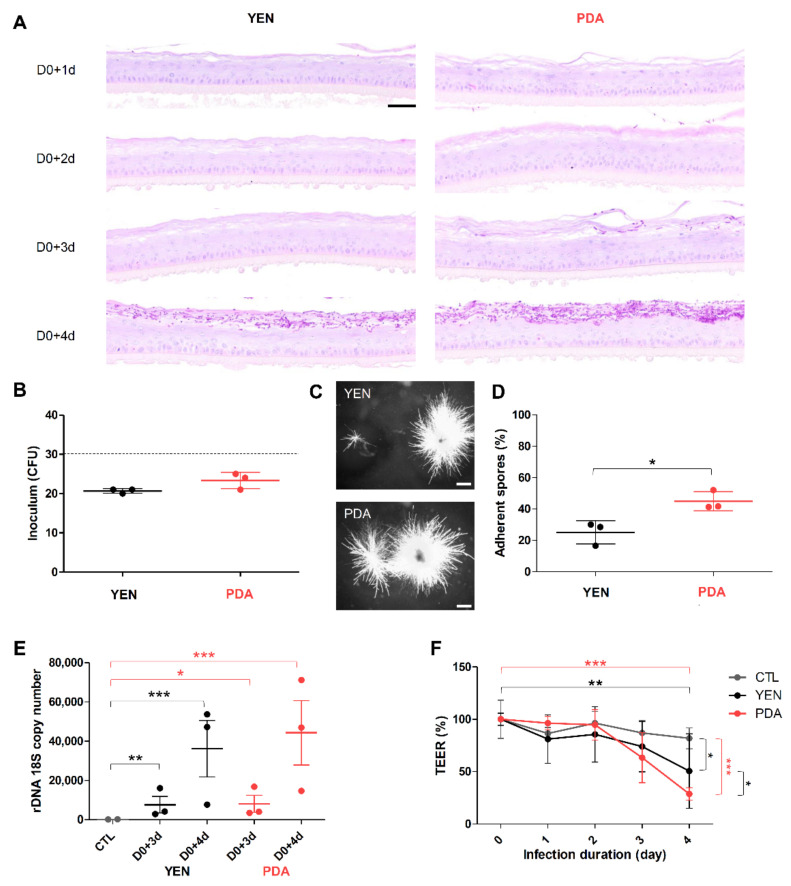
*T. benhamiae* IHEM 20163 infection on reconstructed human epidermis (RHE). RHE were infected on day zero (D0) by topical addition of inoculum consisting in suspensions of *T. benhamiae* IHEM 20163 spores recovered after 21 days of incubation over Yeast Extract Nitrogen agar (YEN) at 30 °C under 12% CO_2_, or after 10 days of incubation over Potato Dextrose Agar (PDA) in the same conditions. The inoculum was adjusted to a density of 30 colony-forming units (CFU) per RHE. (**A**) Histological sections were prepared from infected RHE analysed one (D0+1d), two (D0+2d), three (D0+3d) or four (D0+4d) days after infection and then stained using the Periodic-acid Schiff (PAS) procedure with α-amylase pre-treatment and hemalun counterstaining. Scale bar = 50 µm. (**B**) Fungal load of inoculum was monitored by counting CFU, the dotted line indicates the expected size of inoculum, and (**C**) the macroscopic aspect of CFU was observed. Scale bars = 1 mm. (**D**) Percentage of spores adhering to the epidermis was determined four hours after infection. (**E**) Infection levels were assessed three (D0+3d) and four (D0+4d) days after infection of RHE using qPCR analysis of the rDNA 18S gene copy numbers of *T. benhamiae* in total DNA extracts prepared from infected RHE (YEN and PDA) or non-infected RHE (CTL). (**F**) Epidermal barrier integrity of RHE during infection was determined by measurements of trans-epithelial electrical resistance (TEER). TEER values are expressed as percentage of values measured before RHE infection (D0). Data analysis: n = 3; means ± SD; ANOVA1 for (**B**,**D**), ANOVA2 for (**E**,**F**); * *p* < 0.05 ** *p* < 0.01 *** *p* < 0.001.

As assessed by morphological analysis, invasion was restricted to the cornified layer, whatever the procedure used for inoculum production (Figure 3A and Figure 4A). Monitoring the fungal load of inoculum by seeding on SAB and subsequently performing CFU counting revealed a number of CFU close to the expected value (i.e., 30 CFU for *T. benhamiae* and 1000 CFU *T. rubrum*) (Figure 3B,C and Figure 4B,C). In contrast, the percentage of adherent spores on the tissue surface after four hours of contact appeared significantly higher when the spores were produced on PDA in comparison with spores produced on YEN (Figure 3D and Figure 4D). The more efficient adherence of spores produced on PDA resulted in a tendency towards a greater infection level quantified in the RHE three days or even more four days after their infection (Figure 3E and Figure 4E), as well as for a major impact on the functional integrity of the epidermal barrier (Figure 3F and Figure 4F). 

#### 3.4.2. Responses of Keratinocytes to Infection Are More Consistent When Spores Are Produced on PDA

**Figure 5:**
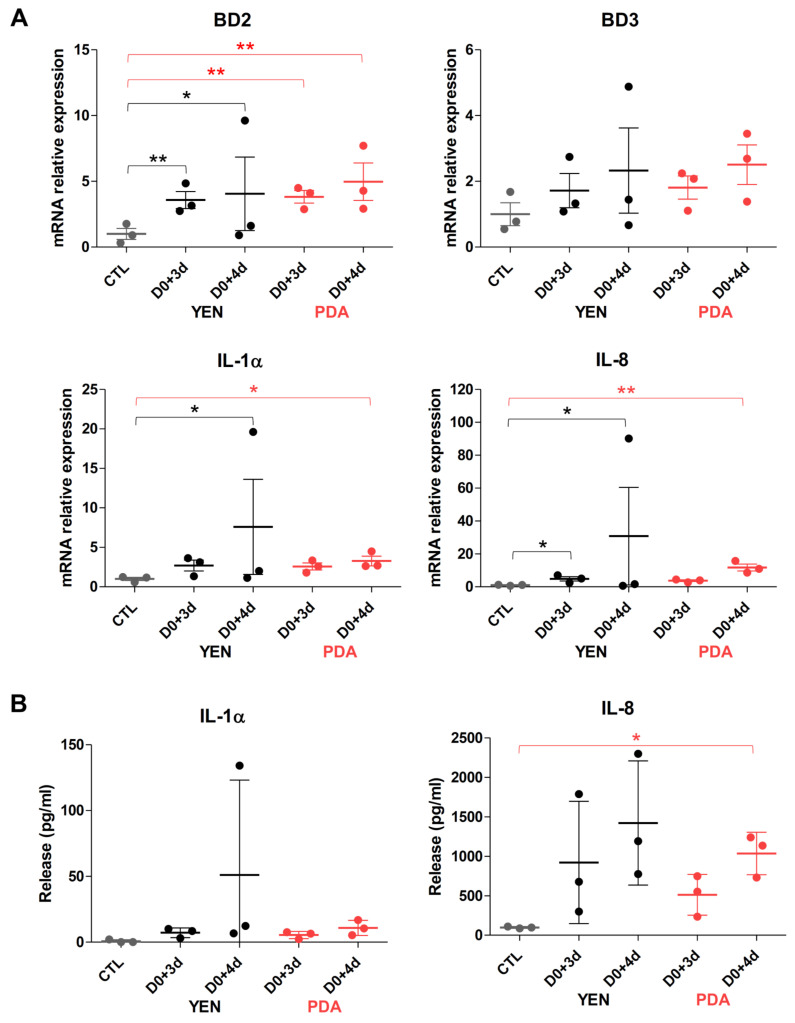
Keratinocytes responses during *T. rubrum* IHEM 13894 infection on reconstructed human epidermis (RHE). RHE were infected on day zero (D0) by topical addition of inoculum consisting in suspensions of *T. rubrum* IHEM 13894 spores recovered after 21 days of incubation over Yeast Extract Nitrogen agar (YEN) at 30 °C under 12% CO_2_, or after 10 days of incubation over Potato Dextrose Agar (PDA) in the same conditions. The inoculum has been adjusted in each case to a density of 1000 colony-forming units (CFU) per RHE. (**A**) mRNA expression of antimicrobial peptides (BD2, BD3) and pro-inflammatory cytokines (IL-1α, IL-8) and (**B**) release of IL-1α and IL-8 by infected RHE, three (D0+3d) and four (D0+4d) days after infection, or by non-infected RHE (CTL) were assessed respectively by RT-qPCR and ELISA. Data analysis: n = 3; means ± SD; ANOVA2; * *p* < 0.05 ** *p* < 0.01.

**Figure 6:**
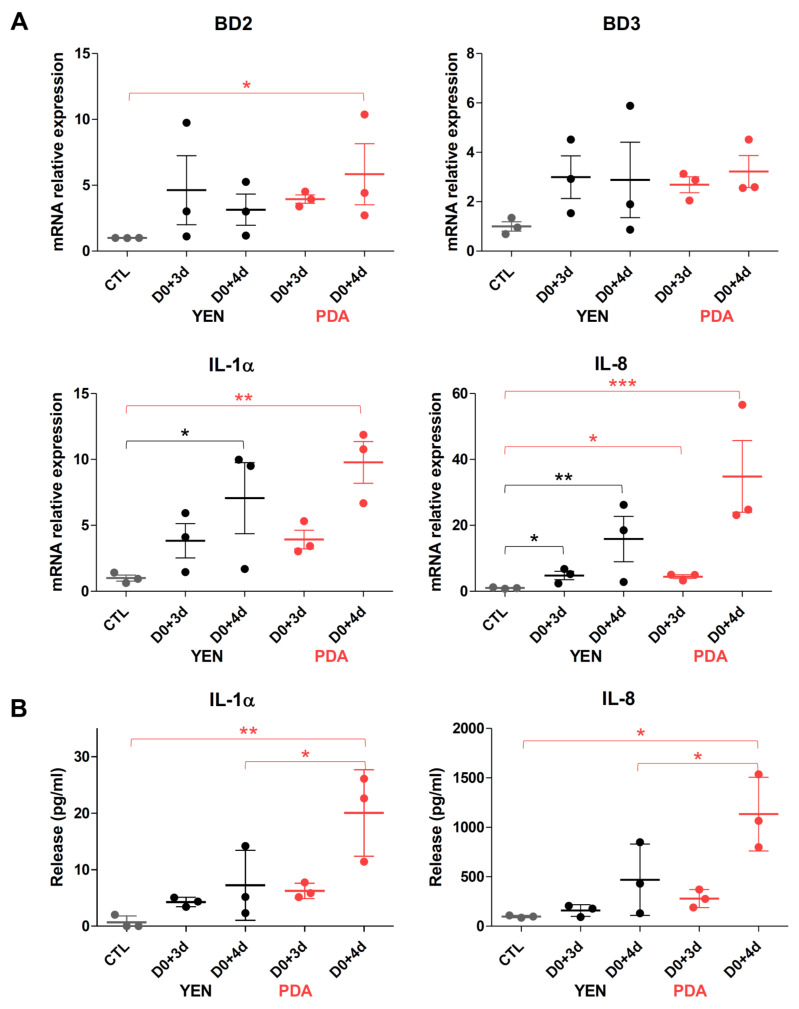
Keratinocytes responses during *T. benhamiae* IHEM 20163 infection on reconstructed human epidermis (RHE). RHE were infected on day zero (D0) by topical addition of inoculum consisting in suspensions of *T. benhamiae* IHEM 20163 spores recovered after 21 days of incubation over Yeast Extract Nitrogen agar (YEN) at 30 °C under 12% CO_2_, or after 10 days of incubation over Potato Dextrose Agar (PDA) in the same conditions. The inoculum has been adjusted in each case to a density of 30 colony-forming units (CFU) per RHE. (**A**) mRNA expression of antimicrobial peptides (BD2, BD3) and pro-inflammatory cytokines (IL-1α, IL-8) and (**B**) release of IL-1α and IL-8 by infected RHE, three (D0+3d) and four (D0+4d) days after infection, or by non-infected RHE (CTL) were assessed respectively by RT-qPCR and ELISA. Data analysis: n = 3; means ± SD; ANOVA2; * *p* < 0.05 ** *p* < 0.01 *** *p* < 0.001.

Keratinocyte responses to the infection of RHE by dermatophytes were assessed through measurements of mRNA expression and release in the culture medium of several pro-inflammatory cytokines (IL-1α, IL-1β, IL-8 and TNFα) and antimicrobial peptides (β-defensin-2 (BD2) and -3 (BD3) and cathelicidin LL-37) (Figure 5, Figure 6 and Figure S11). Since a previous report from our team has shown that keratinocyte responses mainly appear near the end of the infection period [29], the expression and release of these factors were monitored on the third and fourth days following infection of the RHE. In accordance with previously obtained results, keratinocytes expressed and released proinflammatory cytokines and antimicrobial peptides mainly on the fourth day following infection, and at similar level whatever the procedure used for spore production, either on YEN or on PDA. However, the variability of keratinocyte responses using the inoculum produced on YEN was very high between replicate assays. This factual observation that has already been reported [29] remains difficult to explain and monitor. In contrast, the reproducibility of the observed keratinocyte responses was improved and quite satisfactory with the inoculum produced on PDA. 

## 4. Discussion

Faced with a still increasing incidence of dermatophytosis [2], together with the emergence of antifungal resistance [10,11,12,13,14], a better understanding of fungal mechanisms involved in the pathogenesis of dermatophytosis, as well as of cellular responses developed by the host, has become crucially awaited in order to rationally define new therapeutic strategies. For this purpose, several in vitro and in vivo infection models have been developed, allowing actual advances in this field [49]. However, unwanted heterogeneity in the production and composition of inocula renders difficult the overall interpretation of observations made on these various models. With the aim to develop some standardized and reproducible procedures for the production of infective spores suitable as inoculum in models, this study characterized several dermatophyte species frequently isolated from human lesions for their growth rate, sporulation, and arthroconidial production in different culture conditions. 

Among the five tested media, i.e., SAB, PDA, YPD, MALT, and YEN depicted as classical media for fungal growth, PDA appeared to improve the growth and sporulation of most dermatophyte strains (e.g., *T. benhamiae* strains and *T. mentagrophytes* TIMM 2789). For other strains, there was no or little differences between media, especially between PDA and SAB, which also efficiently allowed dermatophyte growth and sporulation (e.g., *T. rubrum* IHEM 13894 and *T. interdigitale* IHEM 00584). Furthermore, the growth of *Microsporum* species, and particularly *M. canis* IHEM 21239, was slightly increased on YEN, in favour of the previous use of this medium to produce fungal suspensions of *M. canis* for infection in in vitro [25] and in vivo [26,27] models. However, in order to standardize the medium used for all species and strains, the PDA appears more appropriate. 

In accordance with the better fungal growth on PDA, spore suspensions produced from this medium showed a high viable fungal concentration (>10^6^ CFU/mL), sufficient for further use in in vitro and in vivo infection models, as based on the most important published models [15,16] and personal observations. Addition of the stirring step before the filtration during the procedure of recovery increased the viable fungal concentration by allowing a better separation of unicellular spores from hyphae. This stirring step can be performed through vortexing the suspension for one minute (“fast procedure” used in this paper), or by a four-hours shaking at 4 °C (“long procedure”) for an even more efficient separation of spores, and thereby a higher viable fungal concentration. Furthermore, a shorter incubation time on PDA, 10 days instead of 21, did not have a negative impact on the viable fungal concentration for *Trichophyton* species, which is an advantage in routine laboratory production. 

*M. audouinii* IHEM 10316 and *T. tonsurans* IHEM 25841 are exceptions, as since very low viable fungal concentrations, of less than 10^4^ and 10^5^ CFU/mL respectively, were obtained for both strains, irrespective of the medium and conditions used, and even after a prolonged incubation of 31 days. Interestingly, the total spore concentration for both these strains appeared to be higher under atmospheric tension compared to incubation under 10% CO_2_ or 12% CO_2_, suggesting that incubation of these strains under lower CO_2_ tension should be an alternative to recover spores to a concentration compatible with their use in infectious models.

Dermatophytes are able to produce two types of spores: micro- and macroconidia resulting from hyphal budding, or arthroconidia derived from hyphal fragmentation. Although arthroconidia are described as natural vectors of dermatophytes [36], they have been less frequently used in experimental models because they remain difficult to produce in culture. Indeed, irregular successes have been described for an efficient in vitro production of arthroconidia [39,40,50,51,52]. Based on previous studies reporting the production of arthroconidia after 10- or 14-day incubation on SAB at 37 °C under 10% CO_2_ [43,44,52], or after 14–21-day incubation on YEN at 30 °C under 12% CO_2_ [25], and on our observation that PDA enhances sporulation, we compared the production of arthroconidia by dermatophytes after 10 or 21 days of incubation on SAB, YEN, or PDA, at 37 °C under 10% CO_2_ or at 30 °C under 12% CO_2_. Testing these conditions is in agreement with previous reports showing that PDA medium and high CO_2_ tension are factors that improve dermatophyte sporulation and arthroconidial production [50,53]. 

In the literature, arthroconidia are described as short-chained or spherical or oval unicellular spores [37,54] and are characterized by the presence of a thicker cell wall when compared to microconidia [55]. In our experience, the morphology of arthroconidia appears highly variable and their identification requires an expert observer, which reinforces the need for molecular tools able to facilitate standardized routine identification of arthroconidia. The proportion of arthroconidia numbered in fungal suspensions recovered after incubation on SAB, PDA and YEN was also highly variable between dermatophytes strains, as well as between the culture medium and condition used, and between replicate assays. In addition, the conditions that enhanced arthroconidial production were not the same as those that allowed a high viable concentration required for subsequent infection in experimental models. Nevertheless, after incubation on PDA at 30 °C under 12% CO_2_, identified as the optimal culture condition for viable fungal concentration, dermatophytes produce arthroconidia with percentages ranging from 1% to 68% for *T. rubrum* IHEM 13894 or 82% for *M. canis* IHEM 21239. The spore suspensions produced in these conditions are therefore “enriched in arthroconidia” compared to suspensions exclusively composed of microconidia and previously used as inoculum in many models [17,18,19,20,21,22,23]. 

Finally, to ensure that spores produced using PDA at 30 °C under 12% CO_2_ are able to adhere to and invade host tissue, and thus constitute an adequate inoculum, we evaluated the development of infection by the anthropophilic *T. rubrum* IHEM 13894 and the zoophilic *T. benhamiae* IHEM 20163 strains using the pre-established model of dermatophytosis on RHE [28]. Spores produced on PDA were able to invade the tissue, leading to tissue damages through impairment of the epidermal barrier, and inducing responses by keratinocytes, as measured by the expression and release of cytokines and antimicrobial peptides. Strangely, the adherence of spores to the RHE surface after four hours of contact appeared to be increased when the inoculum was produced on PDA compared to the inoculum produced by the conventional method used for the design of the model (i.e., 21 days of incubation on YEN [28]). In addition, the measured responses of keratinocytes, which were pretty variable when spores were produced on YEN, were more reproducible with spores produced on PDA. 

In conclusion, this study proposes a standardized procedure for the production of infective spores for use as routine inoculum in experimental models of dermatophytosis. This procedure, summarized in Table 5, allows the recovery of fungal suspensions made of unicellular spores, microconidia but also arthroconidia, at a viable concentration sufficient for infection in in vitro and in vivo models. Standardization of the inocula used in the different experimental models developed by research teams worldwide will facilitate reproducibility and the comparison of observations, leading to more uniform results, which will ultimately improve knowledge of the pathogenesis of dermatophytosis. 

## Figures and Tables

**Table 1:** Dermatophyte isolates analysed in this study. Information on each isolate was retrieved from the online catalogue of Belgian Coordinated Collections of Micro-organisms (BCCM)/Institute of Hygiene and Epidemiology-Mycology section (IHEM; https://bccm.belspo.be/catalogues/ihem-catalogue-search; accessed on 1 September 2021).

Genus—Species— Isolate Number	Isolation			
	Year	Lesion	Host	Country
***Microsporum***				
*M. audouinii* IHEM 10316	1997	Tinea capitis	Human	Belgium
*M. canis* IHEM 22957	2009	Tinea	Cat	Belgium
*M. canis* IHEM 22958	2009	Tinea	Cat	Belgium
*M. canis* IHEM 21239	2005	Tinea	Cat	Belgium
***Trichophyton***				
*T. benhamiae* IHEM 20163	2002	Tinea corporis	Human	Switzerland
*T. benhamiae* IHEM 20161	2002	Tinea faciei	Human	Switzerland
*T. interdigitale* IHEM 00584	1981	Tinea pedis	Human	Belgium
*T. mentagrophytes* IHEM 22740	2008	Skin	Human	Switzerland
*T. mentagrophytes* IHEM 22733	2008	Tinea	Cat	Switzerland
*T. mentagrophytes* IHEM 25841	2012	Skin and hairs	Dog	Belgium
*T. mentagrophytes* TIMM 2789 ^1^				
*T. rubrum* IHEM 13894	1985	Tinea cruris and onychomycosis	Human	Democratic Republic of the Congo
*T. tonsurans* IHEM 24958	2008		Human	Belgium

^1^ The strain *T. mentagrophytes* TIMM 2789 was gracefully supplied by T. Yamada from the Institute of Medical Mycology, Teikyo University [45].

**Table 2:** Composition of agar media used in this study.

Composition in g/L	Sabouraud (SAB)	Potato Dextrose Agar (PDA)	Yeast Peptone Dextrose (YPD)	Malt Medium (MALT)	Yeast Extract Nitrogen (YEN)
Tryptone ^1^	10		20	6	10
Glucose ^2^	20		20		
Potato dextrose agar ^3^		39			
Malt extract ^4^				20	
Yeastextract ^5^			10		20
Agar ^6^	20	5	20	20	20
pH	7.5 ± 0.3	5.4 ± 0.5	7.5 ± 0.1	6.1 ± 0.2	7.3 ± 0.2

^1^ Tryptone (peptone from casein) cat. no. 84610.0500; VWR Chemicals, Radnor, PA, USA; ^2^ D(+)-Glucose monohydrate, + de 99 %, extra pur cat. no. 450740010; Acros-organics B.V.B.A, Fisher Scientific, Fair Lawn, NJ, USA; ^3^ Potato dextrose agar cat. no. 110130; Merck KGaA, Darmstadt, Germany; ^4^ Malt Extract cat. no. 1.05391.0500; Merck KGaA, Darmstadt, Germany; ^5^ Yeast extract cat. no. 84601.0500; VWR Chemicals, Radnor, PA, USA; ^6^ Agar cat. no. BP-1423-2; VWR Chemicals, Radnor, PA, USA.

**Table 3:** Sequences of primer pairs used for RT-qPCR.

Gene Symbol	Forward Primer	Reverse Primer
BD2	5′-ATCAGCCATGAGGGTCTTGT-3′	5′-GAGACCACAGGTGCCAATTT-3′
BD3	5′-TCCAGGTCATGGAGGAATCAT-3′	5′-CGAGCACTTGCCGATCTGT-3′
IL-1α	5′-AACCAGTGCTGCTGAAGGAGAT-3′	5′-TGGTCTCACTACCTGTGATGGTTT-3′
IL-1β	5′-TCCCCAGCCCTTTTGTTGA-3′	5′-TTAGAACCAAATGTGGCCGTG-3′
IL-8	5′-GCAGAGGGTTGTGGAGAAGTTT-3′	5′-TTGGATACCACAGAGAATGAATTTTT-3′
LL-37	5′-CCAGGACGACACAGCAGTCA-3′	5′-CTTCACCAGCCCGTCCTTC-3′
RPLP0	5′-ATCAACGGGTACAAACGAGTC-3′	5′-CAGATGGATCAGCCAAGAAGG-3′
TNFα	5′-GAGGCCAAGCCCTGGTATG-3′	5′-CGGGCCGATTGATCTCAGC-3′

**Table 4:** Minimum and maximum proportions of arthroconidia produced after 10, 21 or 31 days of incubation on PDA at 30 °C under 12% CO_2_.

Genus—Species— Isolate Number	Incubation Duration (Days)	Proportion of Arthroconidia (%)	
		Min	Max
***Microsporum***			
*M. audouinii* IHEM 10316	10	nd ^1^	nd
	21	nd	nd
	31	nd	67
*M. canis* IHEM 22957	21	nd	17
*M. canis* IHEM 22958	21	3	67
*M. canis* IHEM 21239	21	1	82
***Trichophyton***			
*T. benhamiae* IHEM 20161	10	1	21
*T. benhamiae* IHEM 20163	10	1	1
*T. interdigitale* IHEM 00584	10	1	9
*T. mentagrophytes* IHEM 22733	10	14	24
*T. mentagrophytes* IHEM 22740	10	30	66
*T. mentagrophytes* IHEM 25841	10	1	15
*T. mentagrophytes* TIMM 2789	10	3	9
*T. rubrum* IHEM 13894	10	4	68
*T. tonsurans* IHEM 24958	10	60	100
	21	29	100
	31	72	93

^1^ A percentage below the limit of detection by Thoma chamber enumeration is indicated as “not determined” (nd).

**Table 5:** Recommendations for a standardized procedure for the production of infective spores useable as inoculum in in vitro and in vivo experimental models.

Step	Procedure	Aim	Comment
Preculture	Incubation for 14 days on SAB at 30 °C	Growth of the starting fungal material	
Recovering of fungal material by scraping and suspension in PBS			
Sporulation	Seeding on PDA and incubation for 10 days at 30 °C under 12% CO_2_	Production of unicellular spores, mainly microconidia, and to a lesser extent arthroconidia	21 days required for *Microsporum* spp.
Recovering of fungal material by scraping and suspension in PBS			
Agitation	Four hours at 4 °C using magnetic stirrer	Separation of unicellular spores from hyphae	One-minute vortexing for fast procedure (reduced efficiency)
Filtration	Through three Miracloth layers	Recovery of unicellular elements only	Short hyphae can sometimes pass through the Miracloth filter
Washing	Centrifugation of the spore suspension at 3000× *g* for 5 min, discarding of the supernatant and resuspension of the pellet in PBS	Elimination of medium residues	Repeat until obtention of a clear supernatant, and at least three times
Final suspension	Resuspension of the pellet in an appropriate volume of PBS depending of the size of the pellet and of the final spore concentration wanted		Store spore suspensions at 4 °C and use within one month

## Supplementary Materials

The following are available online at https://www.mdpi.com/article/10.3390/jof7121029/s1, Figure S1: Impact of the culture medium on fungal growth, Figure S2: Impact of the culture medium on the radial growth of dermatophytes, Figure S3: Macroscopic aspect of *T. rubrum* IHEM 13894 and of *T. benhamiae* IHEM 20163 colonies, Figure S4: Impact of medium on sporulation of dermatophytes, Figure S5: Viable fungal concentration in infective spore suspensions of *Trichophyton* species, Figure S6: Viable fungal concentration in infective spore suspensions of *Trichophyton mentagrophytes* strains, Figure S7: Viable fungal concentration in infective spore suspensions of *Microsporum* species, Figure S8: Morphology of dermatophyte arthroconidia, Figure S9: Correlation between viable fungal concentration and percentage of arthroconidia in spore suspensions recovered under several culture conditions, Figure S10: Microscopic aspect of spore suspensions of *T. rubrum* IHEM 13894 and of *T. benhamiae* IHEM 20163, Figure S11: Keratinocyte responses during (A) *T. rubrum* IHEM 13894 or (B) *T. benhamiae* IHEM 20163 infection on reconstructed human epidermis (RHE).
