# Supplementary material for: Towards a Standardized Procedure for the Production of Infective Spores to Study the Pathogenesis of Dermatophytosis
Source: J Fungi (Basel). 2021 Nov 30;7(12):1029. doi: 10.3390/jof7121029 (PMC8709344; doi:10.3390/jof7121029)
Supplement: Supplementary file 1 [file jof-07-01029-s001.zip › Faway, Staerck et al_Revised Supplementary materials.pdf]

Journal of *Fungi*

MDPI

Supplementary materials

Towards a Standardized Procedure for the Production of Infective Spores to Study the Pathogenesis of Dermatophytosis

Emilie Faway [1,†], Cindy Staerck [2,†], Célya Danzelle [2], Sophie Vroomen [2], Christel Courtain [1], Bernard Mignon [2,*,‡] and Yves Poumay [1,*,‡]

1 Molecular Physiology Research Unit, NAmur Research Institute for LIfe Sciences (URPHYM-NARILIS), Faculty of Medicine, University of Namur, 5000 Namur, Belgium; emilie.faway@unamur.be (E.F.); christel.courtain@student.unamur.be (C.C.)

2 Fundamental and Applied Research for Animals & Health (FARAH), Faculty of Veterinary Medicine, University of Liège, 4000 Liège, Belgium; cindy.staerck@uliege.be (C.S.); celya.danzelle@uliege.be (C.D.); sophie.vroomen@uliege.be (S.V.)

* Correspondence: bmignon@uliege.be (B.M.); yves.poumay@unamur.be (Y.P.); Tel.: +32-4-366-40-99 (B.M.); +32-81-72-42-57 (Y.P.)

† These authors contributed equally to this work.

‡ These authors contributed equally to this work.

Abstract: Dermatophytoses are superficial infections of human and animal keratinized tissues caused by filamentous fungi named dermatophytes. Because of a high and increasing incidence, as well as the emergence of antifungal resistance, a better understanding of mechanisms involved in adhesion and invasion by dermatophytes is required for further development of new therapeutic strategies. In the last years, several *in vitro* and *in vivo* models have emerged to study dermatophytosis pathogenesis. However, the procedures used for the culture of fungi are quite different, leading to a highly variable composition of inoculum for these models (microconidia, arthroconidia, hyphae), thus rendering difficult the global interpretation of observations. We hereby optimized growth conditions, including medium, temperature, atmosphere and duration of culture, to improve the sporulation and viability and to favour the production of arthroconidia of several dermatophyte species, including *Trichophyton rubrum* and *Trichophyton benhamiae*. The resulting suspensions were then used as inoculum to infect reconstructed human epidermis in order to validate their ability to adhere on and to invade host tissues. By this way, this paper provides recommendations for dermatophytes culture and paves the way towards a standardized procedure for the production of infective spores usable in *in vitro* and *in vivo* experimental models.

Keywords: dermatophytosis; dermatophytes; infective spores; arthroconidia; microconidia; reconstructed human epidermis; dermatophyte pathogenesis; experimental models; *Trichophyton*; *Microsporum*

Citation: Faway, E.; Staerck, C.; Danzelle, C.; Vroomen, S.; Courtain, C.; Mignon, B.; Poumay, Y. Towards a Standardized Procedure for the Production of Infective Spores to Study the Pathogenesis of Dermatophytosis. *J. Fungi* **2021**, *7*, 1029. https://doi.org/10.3390/jof7121029

Academic Editor: Anuradha Chowdhary

Received: 28 October 2021
Accepted: 26 November 2021
Published: 30 November 2021

Publisher's Note: MDPI stays neutral with regard to jurisdictional claims in published maps and institutional affiliations.

Copyright: © 2021 by the authors. Licensee MDPI, Basel, Switzerland. This article is an open access article distributed under the terms and conditions of the Creative Commons Attribution (CC BY) license (http://creativecommons.org/licenses/by/4.0/).

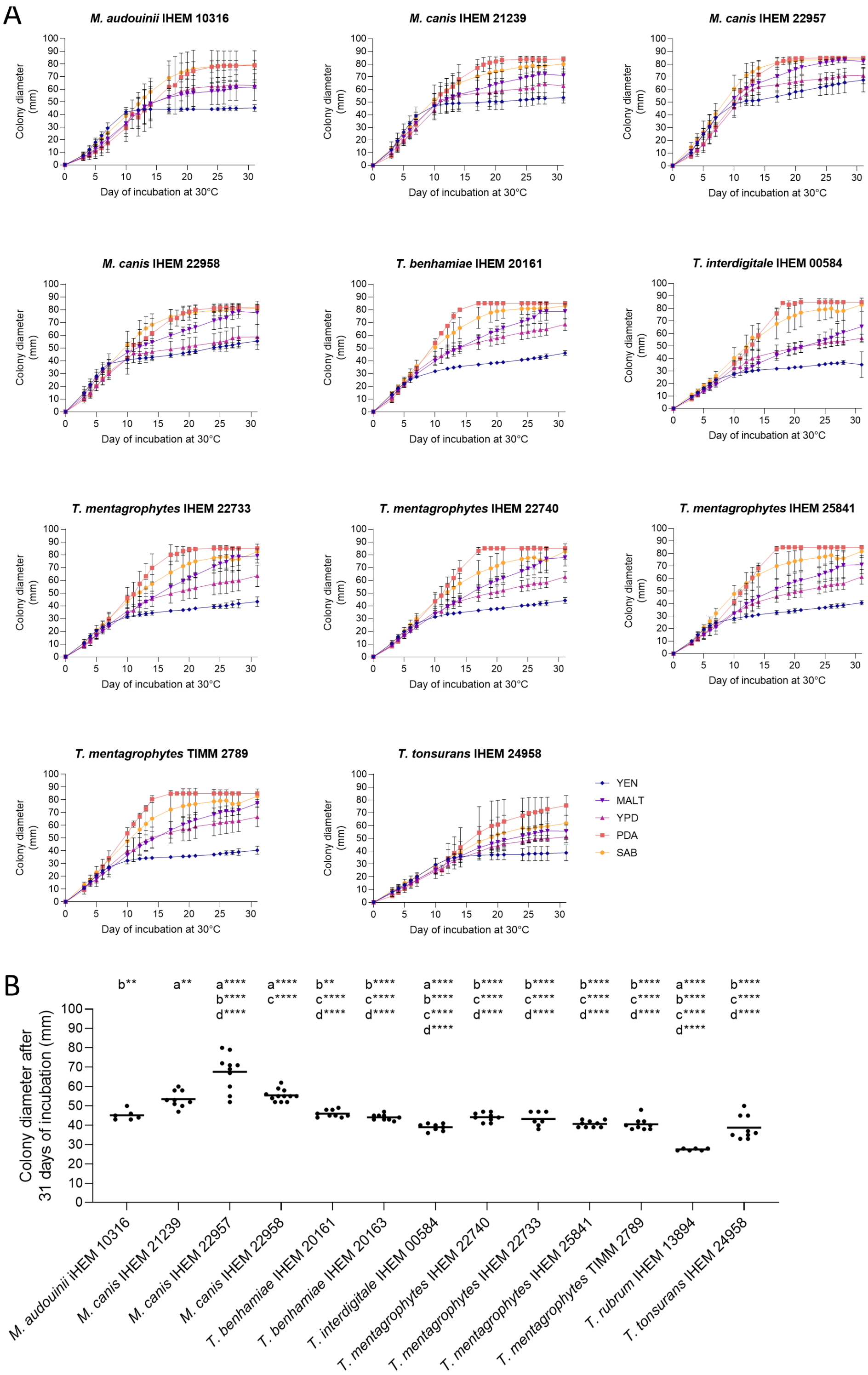

Figure S1: Impact of the culture medium on fungal growth. Dermatophyte strains were seeded over five agar media, Sabouraud (SAB), Potato Dextrose Agar (PDA), Yeast Peptone Dextrose (YPD), Malt medium (MALT) or Yeast Extract Nitrogen (YEN), and grown at 30°C for 31 days. **(A)** Diameter of fungal colonies was measured daily. **(B)** Diameter of fungal colonies was measured after 31 days of incubation on YEN at 30°C for each dermatophyte strain and compared to the colony diameter of *Microsporum* strains, *M. audouinii* IHEM 10316 (a), *M. canis* IHEM 21239 (b), *M. canis* IHEM 22957 (c) and *M. canis* IHEM 22958 (d). Data analysis: n≥ 3; means ± SD for A, means and individual values for B; ANOVA2; *p<0.05 **p<0.01 ***p<0.001 ****p<0.0001.

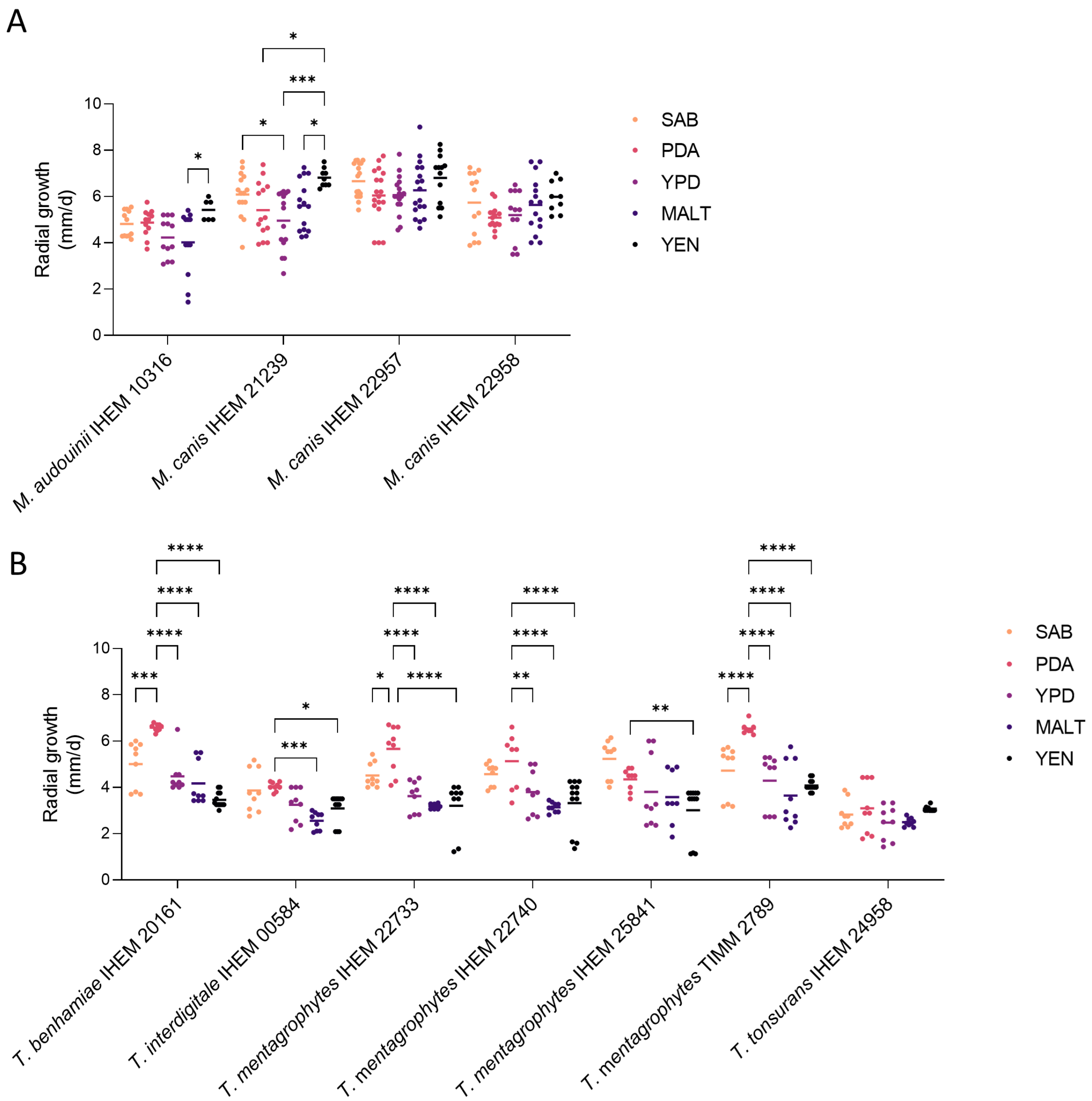

Figure S2: Impact of the culture medium on the radial growth of dermatophytes. Dermatophyte strains were seeded over five agar media, Sabouraud (SAB), Potato Dextrose Agar (PDA), Yeast Peptone Dextrose (YPD), Malt medium (MALT) or Yeast Extract Nitrogen (YEN), and incubated at 30°C for 31 days. Radial growth of **(A)** *Microsporum* and **(B)** *Trichophyton* strains was determined during the exponential phase of growth. Data analysis: n≥ 3; means and individual values; ANOVA2; *p<0.05 **p<0.01 ***p<0.001 ****p<0.0001.

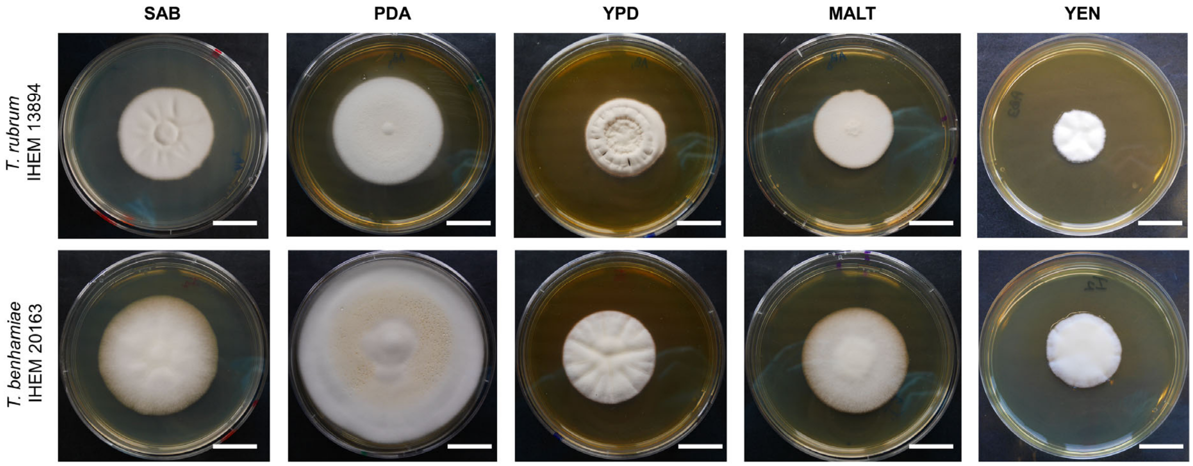

Figure S3: Macroscopic aspect of *T. rubrum* IHEM 13894 and of *T. benhamiae* IHEM 20163 colonies. Dermatophytes were spotted on the centre of the five agar plates, *i.e.* Sabouraud (SAB), Potato Dextrose Agar (PDA), Yeast Peptone Dextrose (YPD), Malt medium (MALT) or Yeast Extract Nitrogen (YEN), and the resulting colonies were observed after 14 days of growth at 30°C. Scale bar = 2 cm.

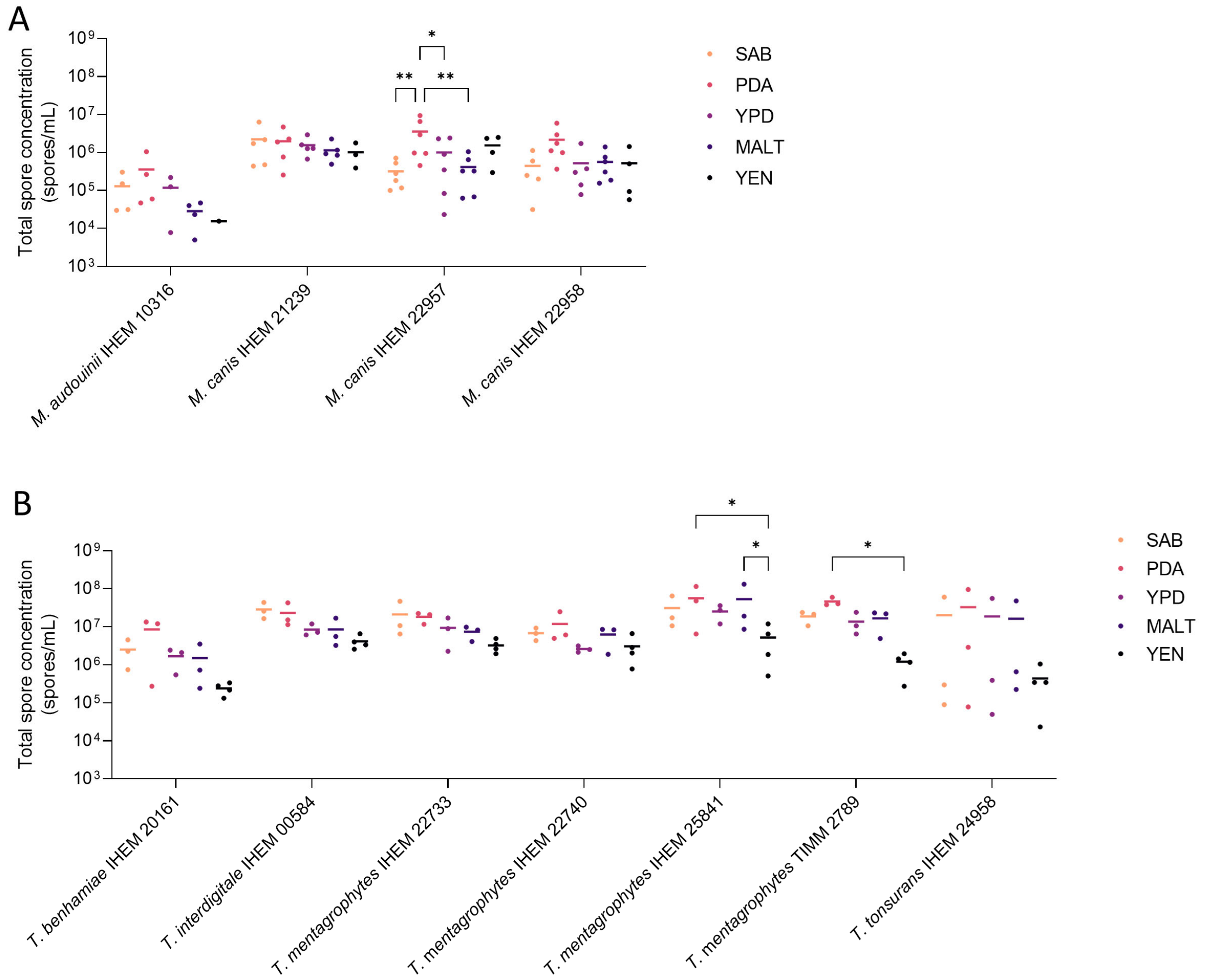

Figure S4: Impact of medium on sporulation of dermatophytes. Dermatophyte strains were seeded over five agar media, Sabouraud (SAB), Potato Dextrose Agar (PDA), Yeast Peptone Dextrose (YPD), Malt medium (MALT) or Yeast Extract Nitrogen (YEN). After 31 days of incubation at 30°C, the fungal material was recovered, suspended in PBS and filtered to obtain fungal suspensions. Total spore concentration in these fungal suspensions was determined by counting using a Thoma chamber under light microscopy, for **(A)** *Microsporum* and **(B)** *Trichophyton* species. Data analysis: n≥ 3; means and individual values; ANOVA2 compared to PDA; *p<0.05 **p<0.01 ***p<0.001 ****p<0.0001.

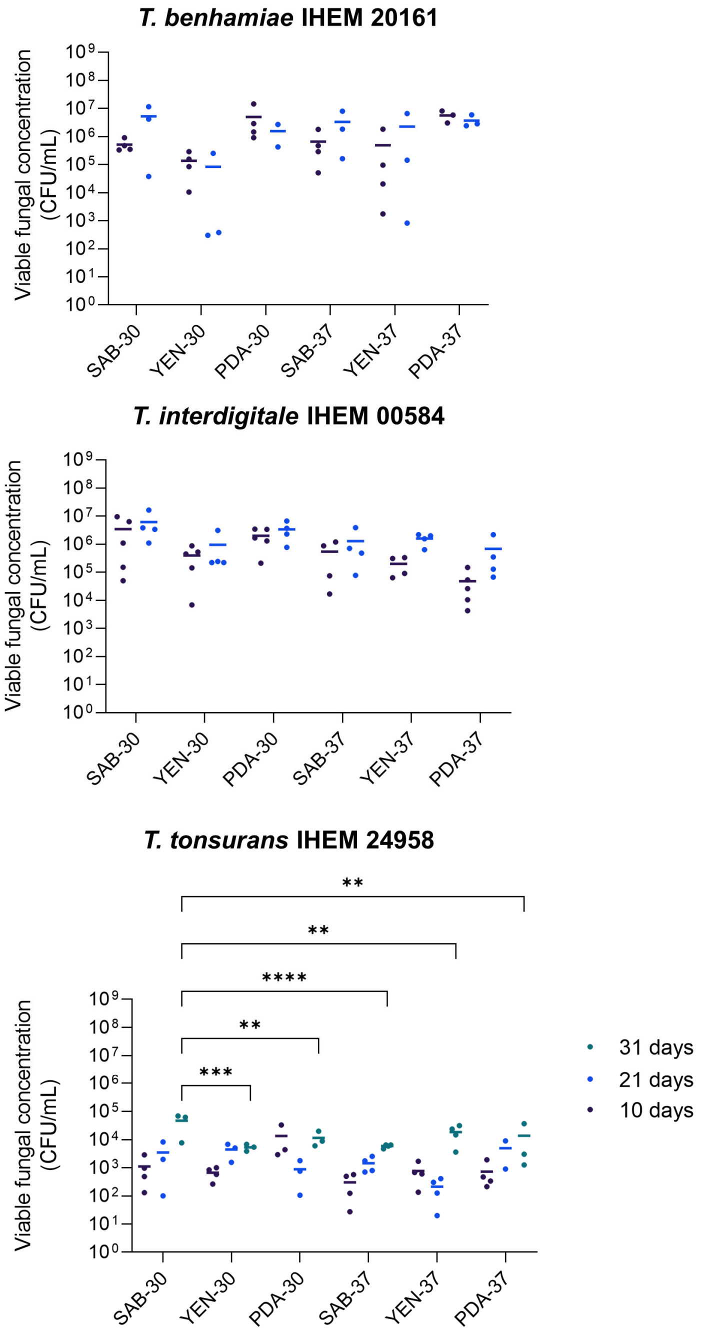

Figure S5: Viable fungal concentration in infective spore suspensions of *Trichophyton* species. To promote sporulation and arthroconidial production by *Trichophyton* species, fungi were seeded over Sabouraud (SAB), Potato Dextrose Agar (PDA) or Yeast Extract Nitrogen (YEN) media, and incubated at 30°C under 12% CO_2 or at 37°C under 10% CO_2, for 10 or 21 days, or 31 days for *T. tonsurans* IHEM 24958. The fungal material was then recovered, suspended in PBS, stirred and filtered to obtain spore suspensions. Viable fungal concentration in these suspensions was determined by counting colony-forming units (CFU). Data analysis: n≥ 3; means and individual values; ANOVA2 compared to PDA-30 (or to SAB-30 for T. tonsurans IHEM 24958); *p<0.05 **p<0.01 ***p<0.001 ****p<0.0001.

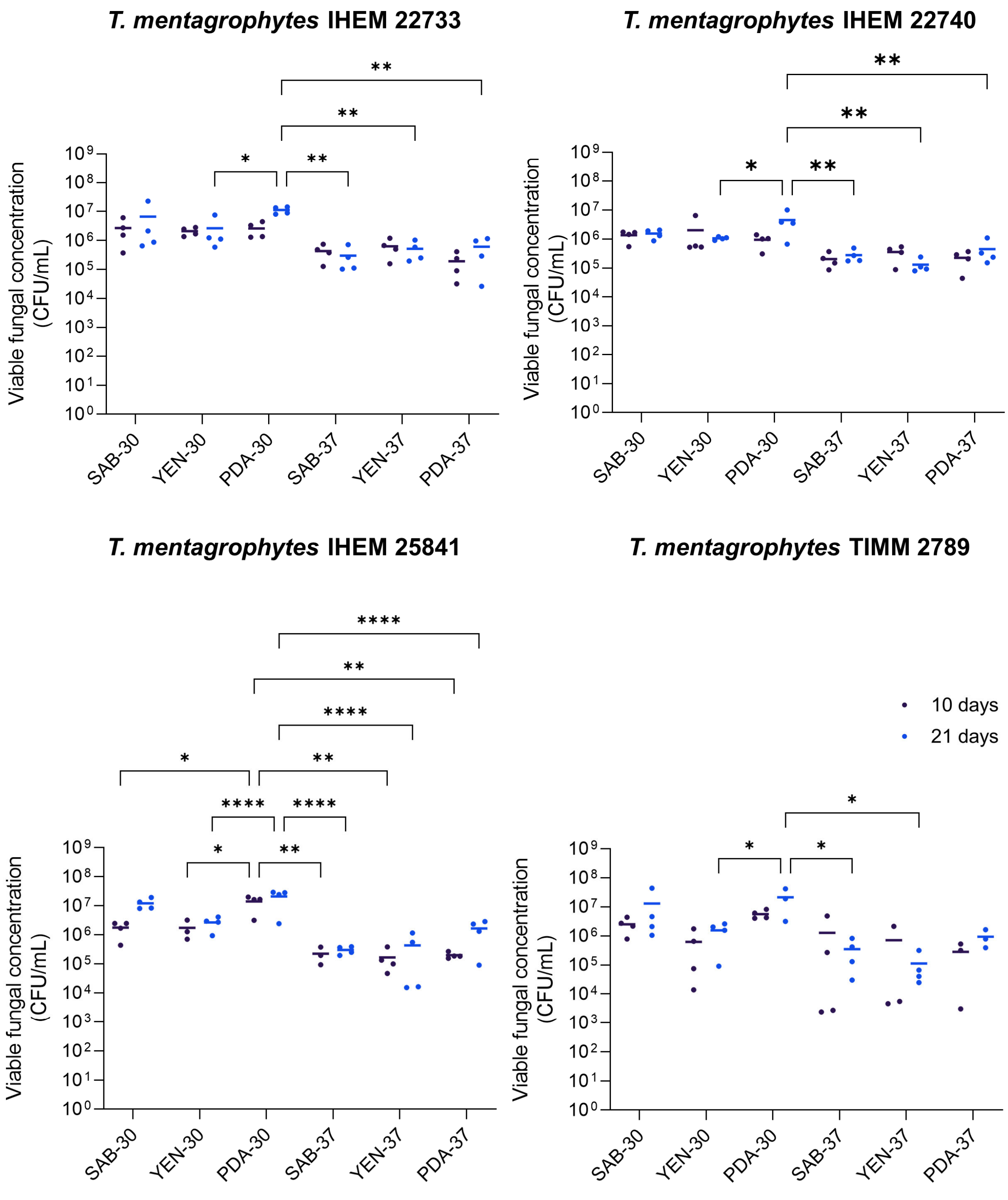

Figure S6: Viable fungal concentration in infective spore suspensions of *Trichophyton mentagrophytes* strains. To promote sporulation and arthroconidial production by *T. mentagrophytes* strains, fungi were seeded over Sabouraud (SAB), Potato Dextrose Agar (PDA) or Yeast Extract Nitrogen (YEN) media, and incubated at 30°C under 12% CO_2 or at 37°C under 10% CO_2, for 10 or 21 days. The fungal material was then recovered, suspended in PBS, stirred and filtered to obtain spore suspensions. Viable fungal concentration in these suspensions was determined by counting colony-forming units (CFU). Data analysis: n≥ 3; means and individual values; ANOVA2 compared to PDA-30 (or to SAB-30 for T. tonsurans IHEM 24958); *p<0.05 **p<0.01 ***p<0.001 ****p<0.0001.

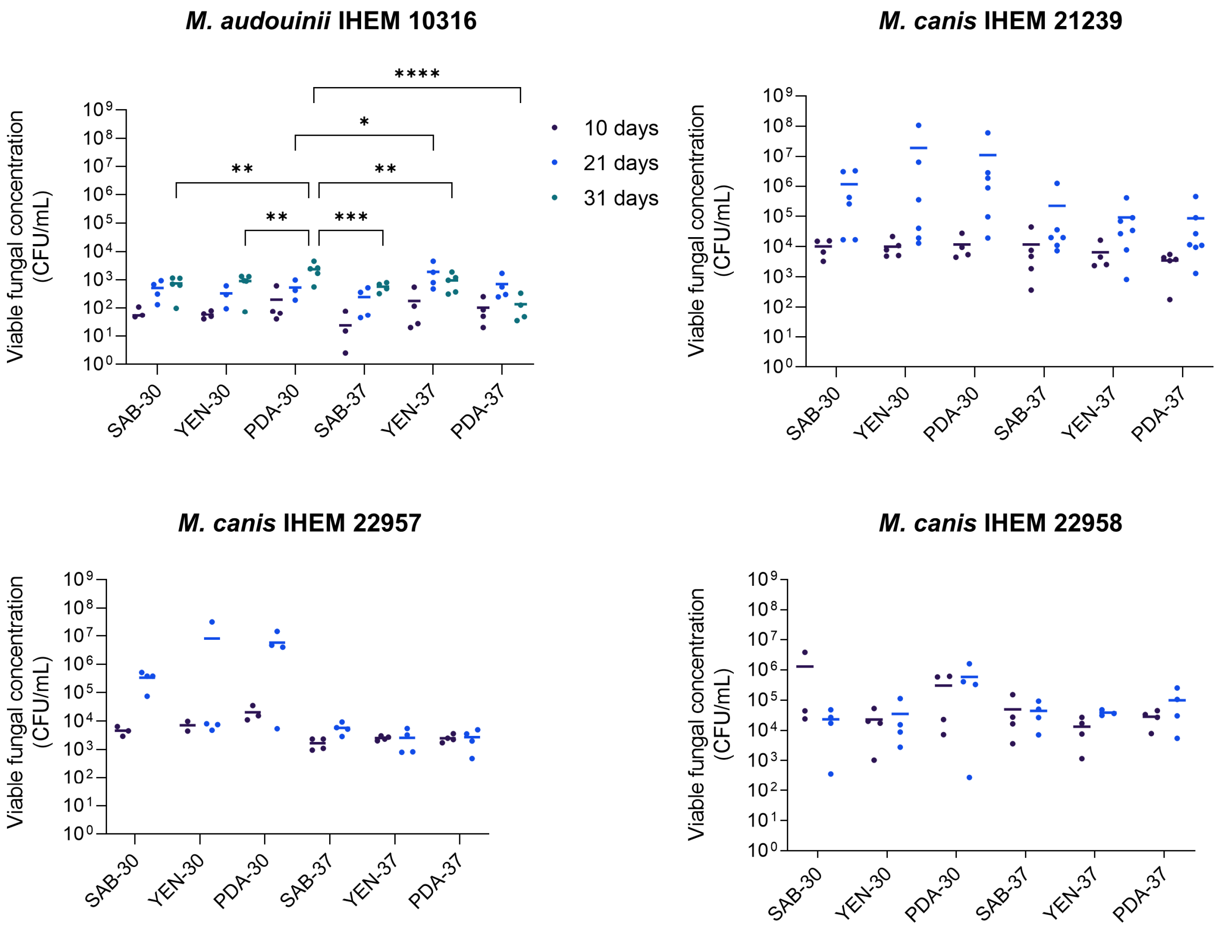

Figure S7: Viable fungal concentration in infective spore suspensions of *Microsporum* species. To promote sporulation and arthroconidial production by *Microsporum* species, fungi were seeded over Sabouraud (SAB), Potato Dextrose Agar (PDA) or Yeast Extract Nitrogen (YEN) media, and incubated at 30°C under 12% CO_2 or at 37°C under 10% CO_2, for 10 or 21 days, or 31 days for *M. audouinii* IHEM 10316. The fungal material was then recovered, suspended in PBS, stirred and filtered to obtain spore suspensions. Viable fungal concentration in these suspensions was determined by counting colony-forming units (CFU). Data analysis: n≥ 3; means and individual values; ANOVA2 compared to PDA-30 (or to SAB-30 for T. tonsurans IHEM 24958); **p*<0.05 ***p*<0.01 ****p*<0.001 *****p*<0.0001.

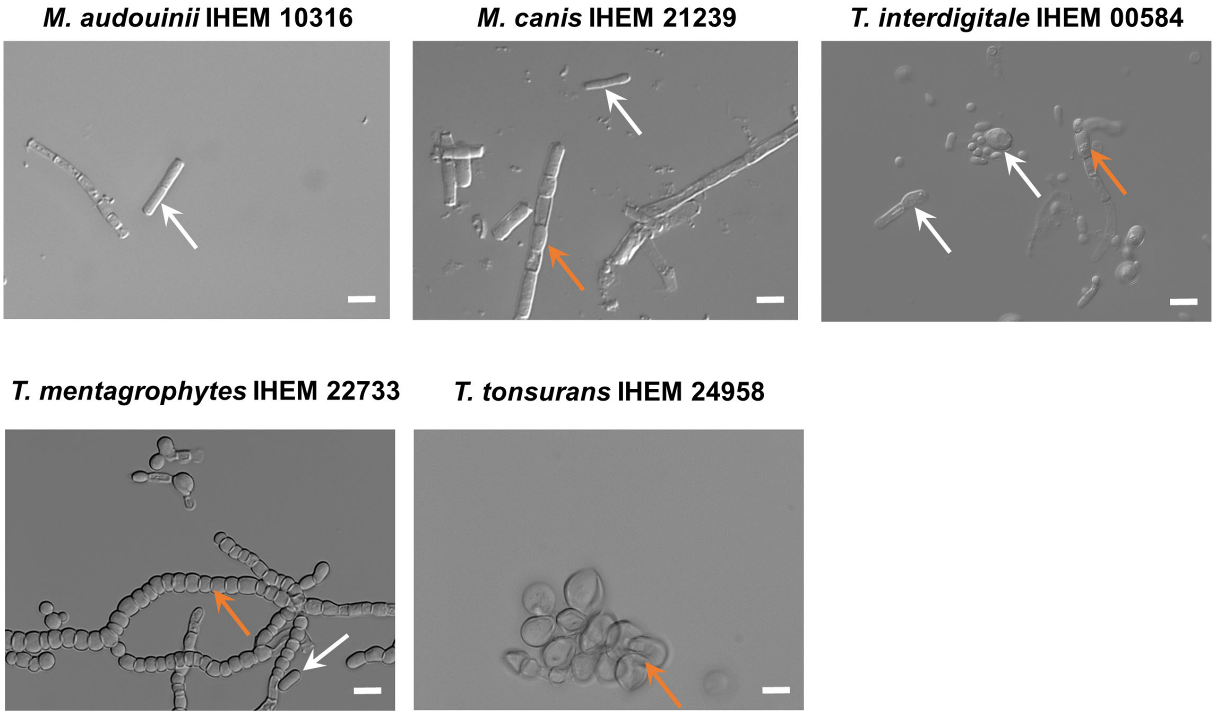

Figure S8: Morphology of dermatophyte arthroconidia. A few microliters of spore suspensions of several dermatophyte species were laid over a microscopic slide and observed under differential interference contrast (DIC) microscopy. Individual arthroconidia released from hyphae are shown by white arrows, whereas orange arrows indicate arthroconidia within hyphae. Scale bar = 10 μm.

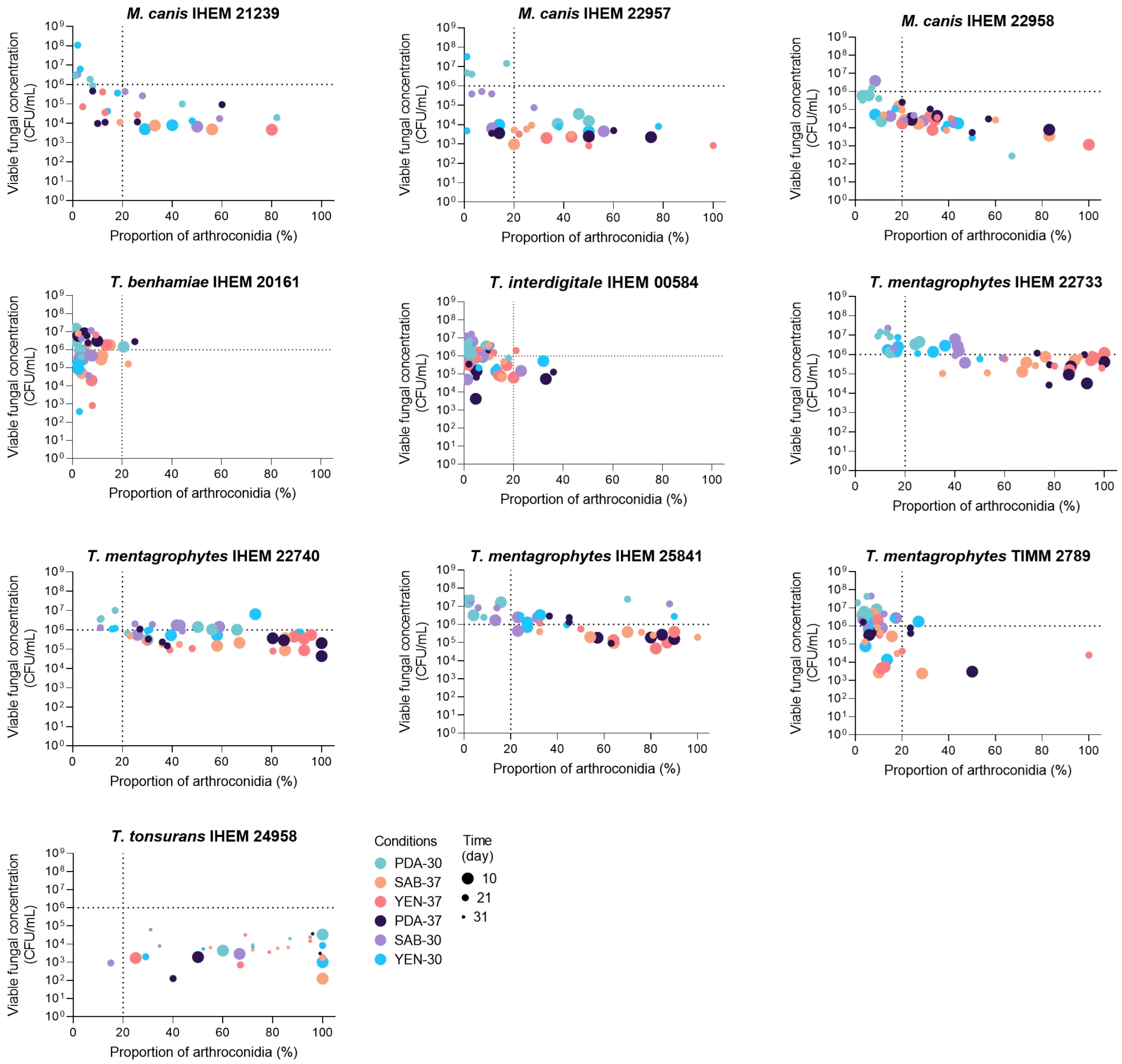

Figure S9: Correlation between viable fungal concentration and percentage of arthroconidia in spore suspensions recovered under several culture conditions. To promote sporulation and arthroconidial production by dermatophytes, fungi were seeded over Sabouraud (SAB), Potato Dextrose Agar (PDA) or Yeast Extract Nitrogen (YEN) media, and incubated at 30°C under 12% CO_2 or at 37°C under 10% CO_2, for 10 or 21 days, or 31 days for *T. tonsurans* IHEM 24958. Viable fungal concentration, measured by colony-forming units (CFU) counting, was correlated with the proportion of arthroconidia relative to the total number of fungal elements, determined by counting using a Thoma chamber under light microscopy. Dotted lines indicate the minimal viable fungal concentration required for further use on experimental models, based on the most important published models [19; 20] and personal observations, as well as the proportion of 20% arthroconidia, arbitrarily chosen as a minimal value for suspensions enriched in arthroconidia. Note that any representation was made for the strain *M. audouinii* IHEM 10316 because the total fungal concentration in recovered suspensions was under the limit of detection of the Thoma chamber and did not allow a proper enumeration of the arthroconidia proportion.

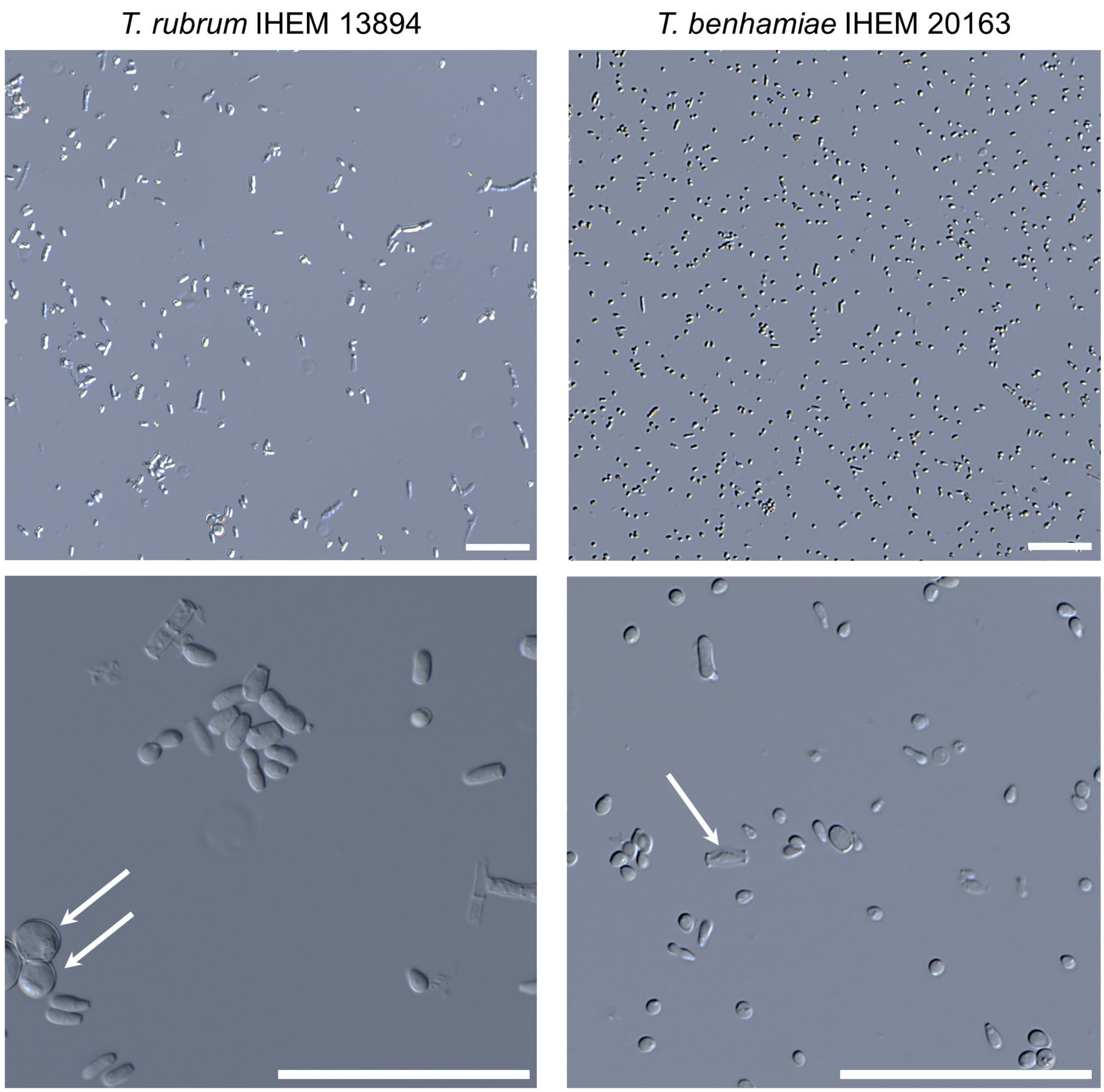

Figure S10: Microscopic aspect of spore suspensions of *T. rubrum* IHEM 13894 and of *T. benhamiae* IHEM 20163. The spore suspensions were recovered after incubation of dermatophytes for 10 days on PDA at 30°C under 12% CO_2, stirring and filtration through three Miracloth layers, and observed using differential interference contrast (DIC) microscopy. Numerous microconidia are observed, as well as few arthroconidia indicated by white arrow. Scale bar = 50 µm.

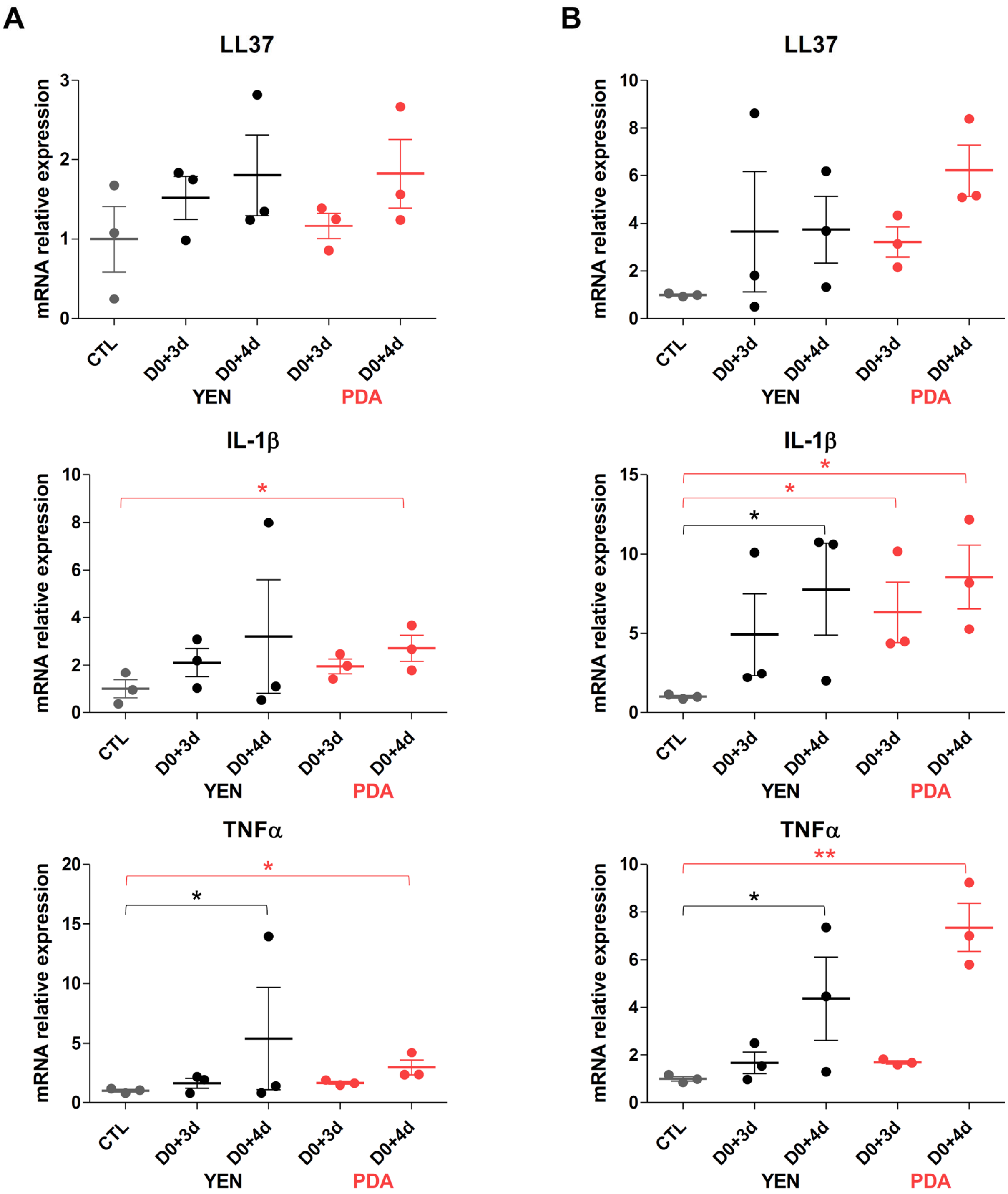

Figure S11: Keratinocyte responses during (A) ***T. rubrum*** **IHEM 13894 or (B)** ***T. benhamiae*** **IHEM 20163 infection on reconstructed human epidermis (RHE).** RHE were infected on day zero (D0) by topical addition of inoculum consisting in spore suspensions recovered after 21 days of incubation over Yeast Extract Nitrogen agar (YEN) at 30°C under 12% CO_2, or after 10 days of incubation over Potato Dextrose Agar (PDA) in the same conditions. The inocula was adjusted to a density of 1000 or 30 colony-forming units (CFU) per RHE respectively for **(A)** *T. rubrum* IHEM 13894 and **(B)** *T. benhamiae* IHEM 20163. mRNA expression of antimicrobial peptides (LL-37) and pro-inflammatory cytokines (IL-1β, TNFα) by keratinocytes of infected RHE, three (D0+3d) and four (D0+4d) days after infection, or by keratinocytes of non-infected RHE (CTL) was assessed by RT-qPCR. Data analysis: n=3; means ± SD; ANOVA2; **p*<0.05 ***p*<0.01 ****p*<0.001 *****p*<0.0001.
