# Supplementary figures and images for: Towards a Standardized Procedure for the Production of Infective Spores to Study the Pathogenesis of Dermatophytosis
Source: J Fungi (Basel). 2021 Nov 30;7(12):1029. doi: 10.3390/jof7121029 (PMC8709344; doi:10.3390/jof7121029)

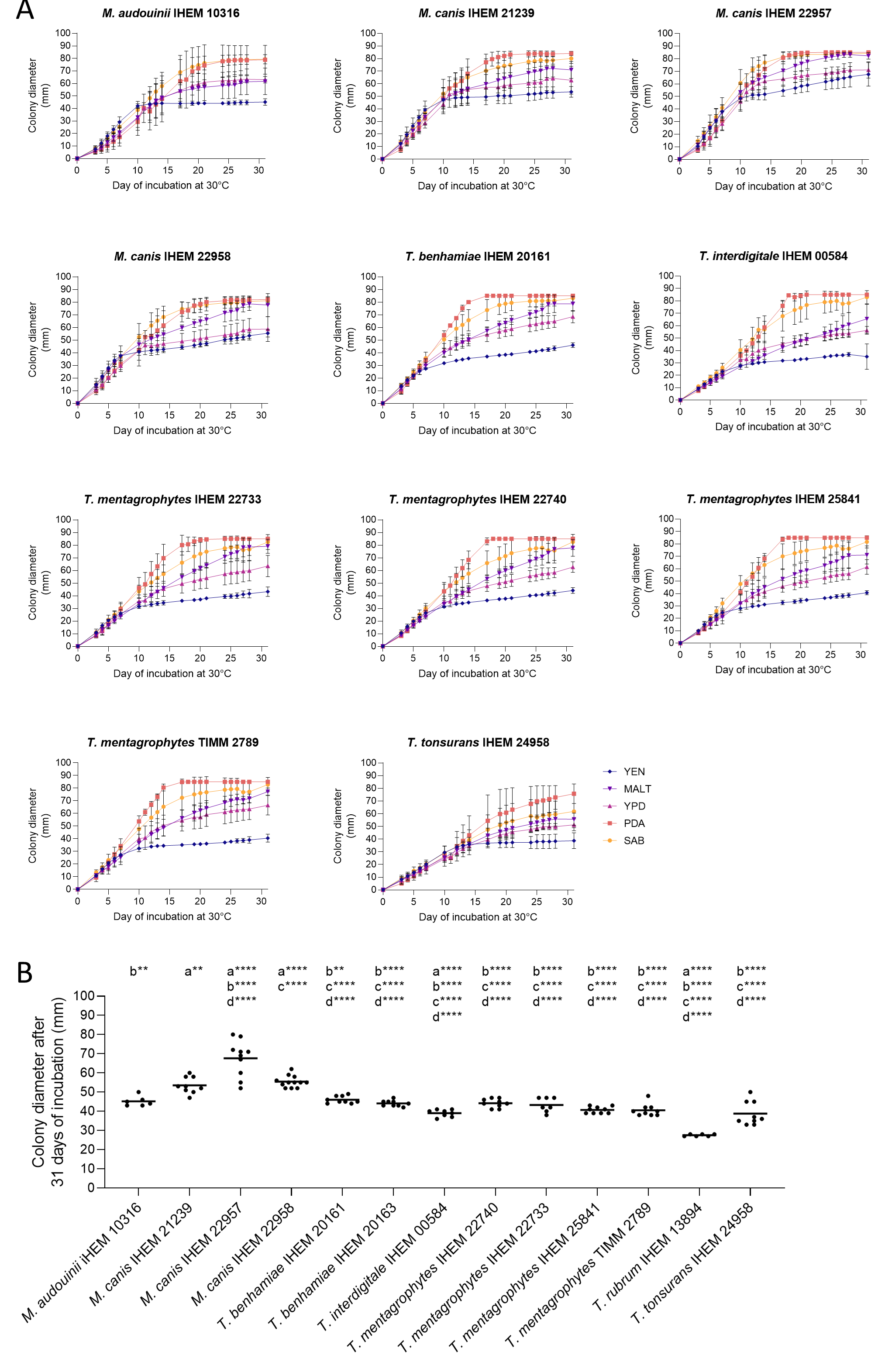

Supplement: Supplementary file 1 [file jof-07-01029-s001.zip › Faway, Staerck et al_Figure S1.tif]

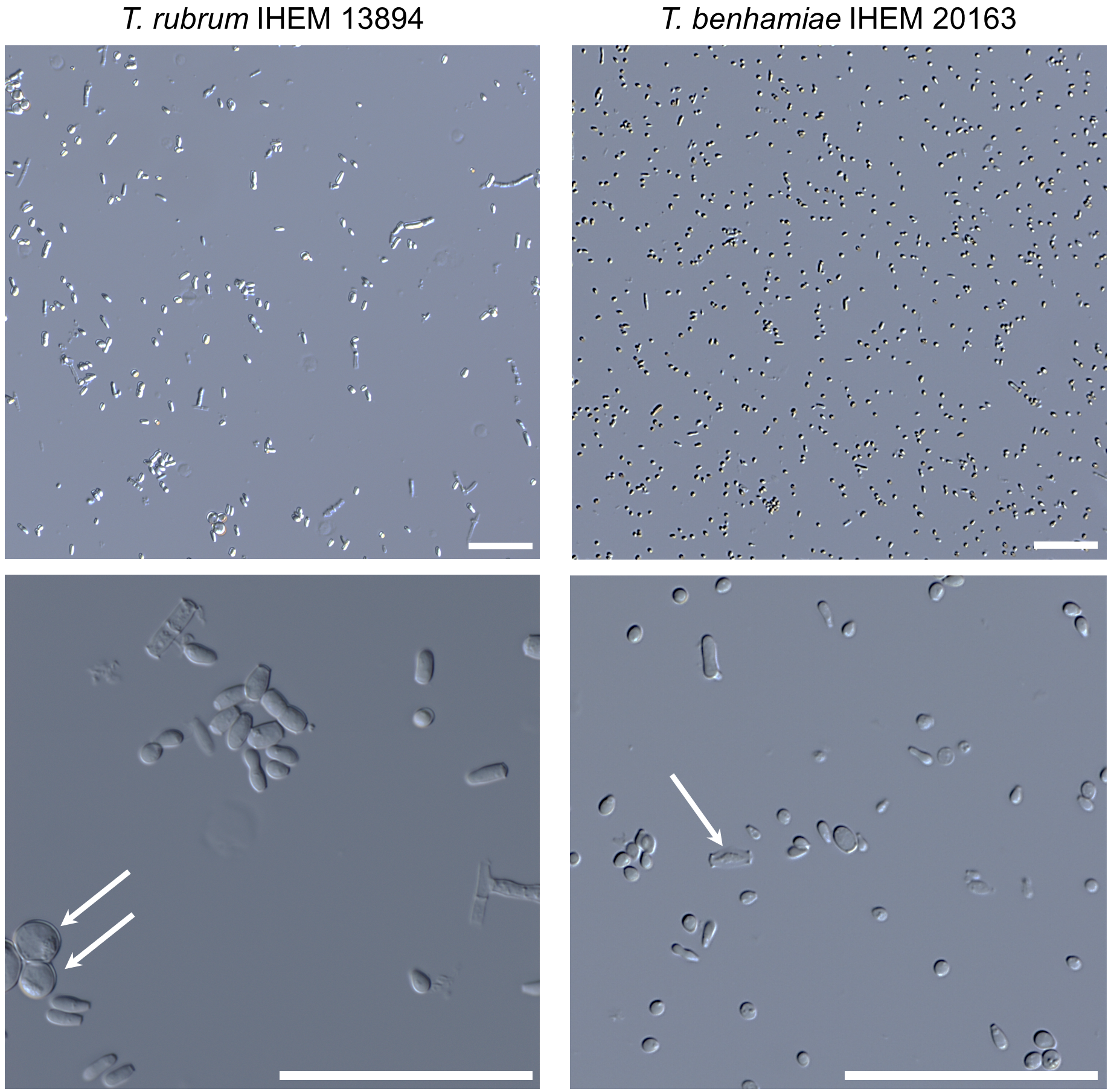

Supplement: Supplementary file 1 [file jof-07-01029-s001.zip › Faway, Staerck et al_Figure S10.tif]

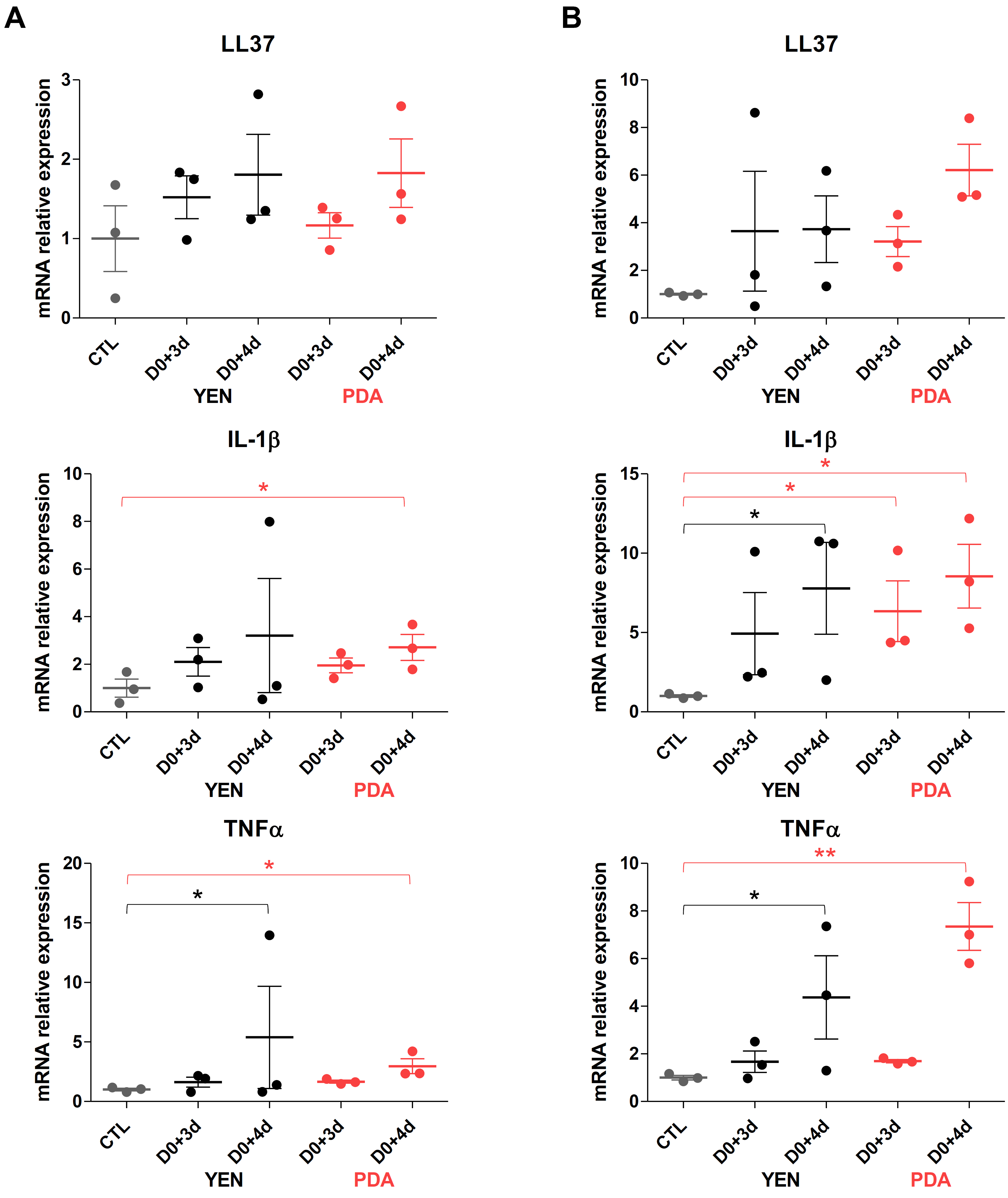

Supplement: Supplementary file 1 [file jof-07-01029-s001.zip › Faway, Staerck et al_Figure S11.tif]

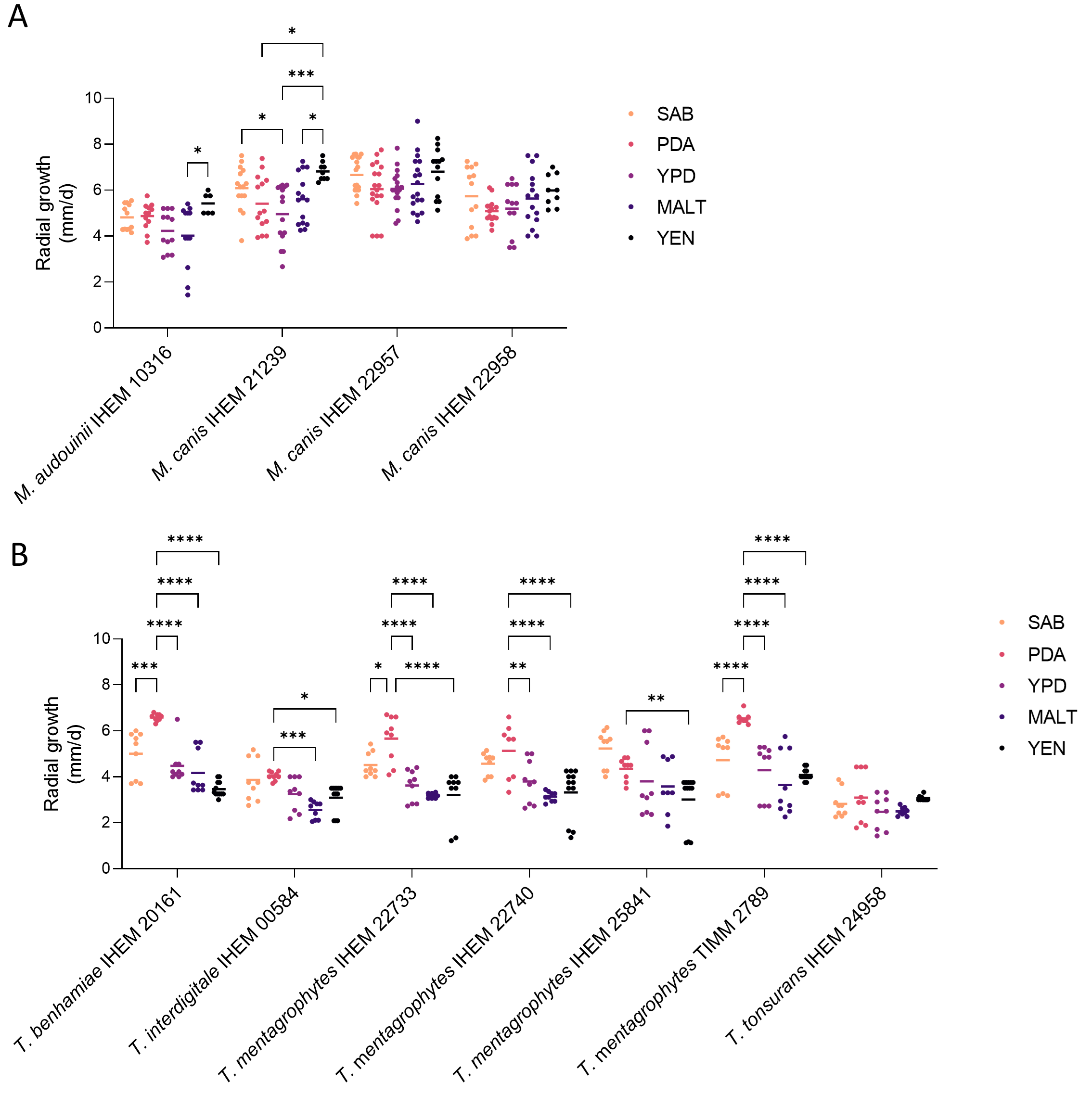

Supplement: Supplementary file 1 [file jof-07-01029-s001.zip › Faway, Staerck et al_Figure S2.tif]

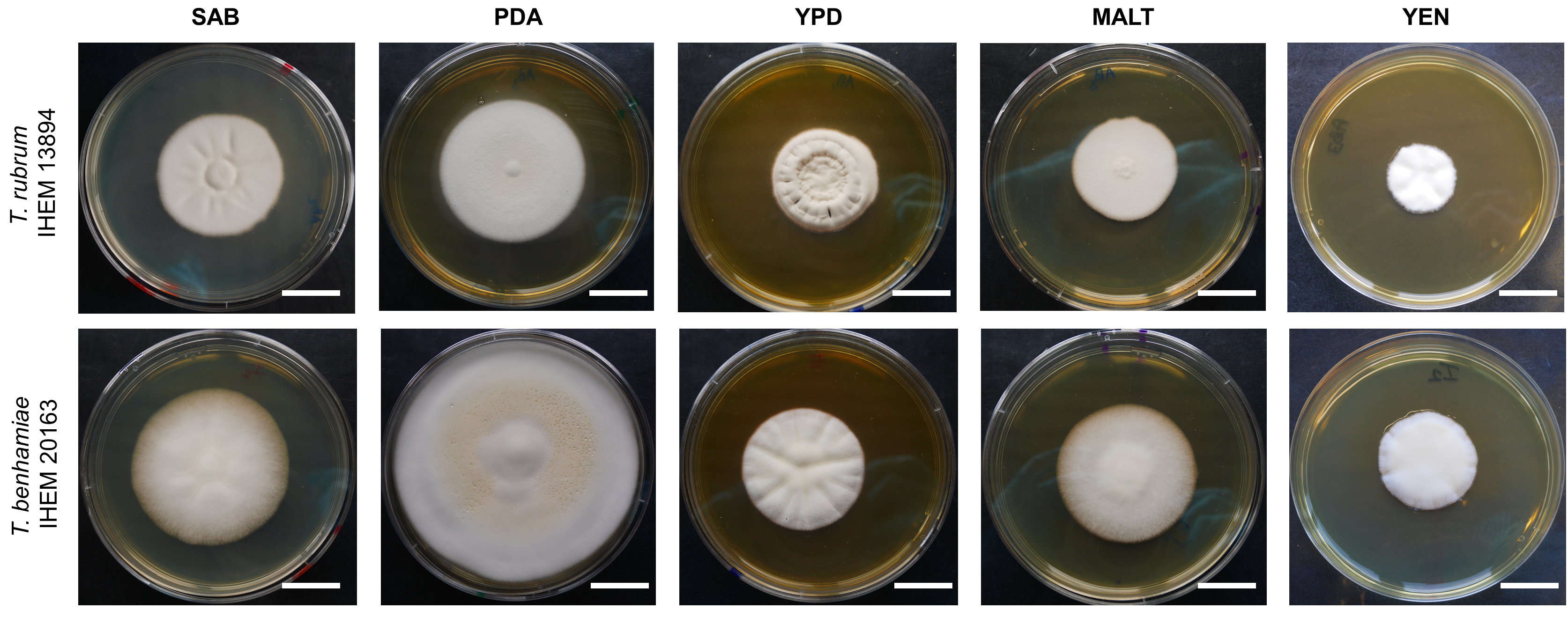

Supplement: Supplementary file 1 [file jof-07-01029-s001.zip › Faway, Staerck et al_Figure S3.tif]

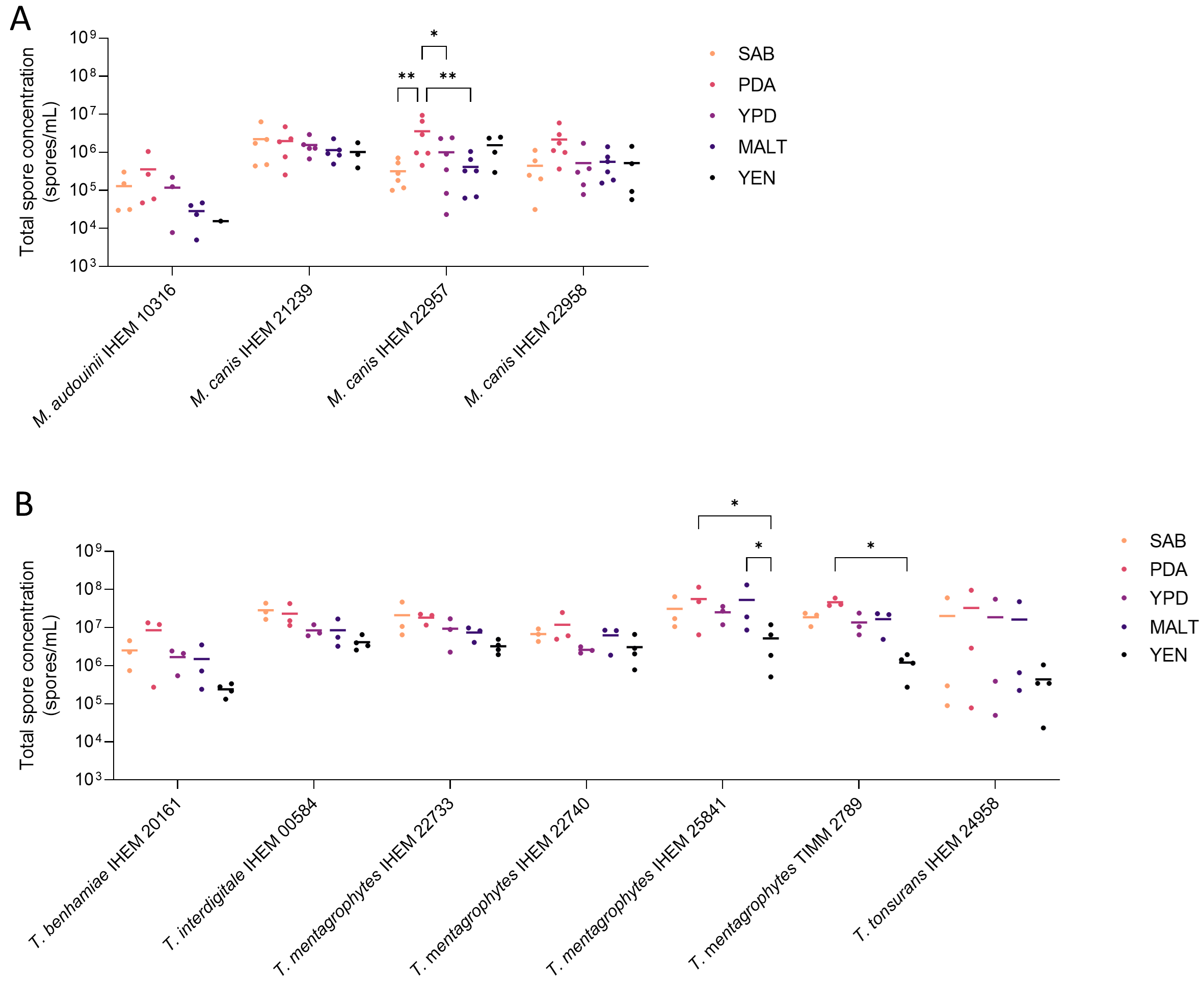

Supplement: Supplementary file 1 [file jof-07-01029-s001.zip › Faway, Staerck et al_Figure S4.tif]

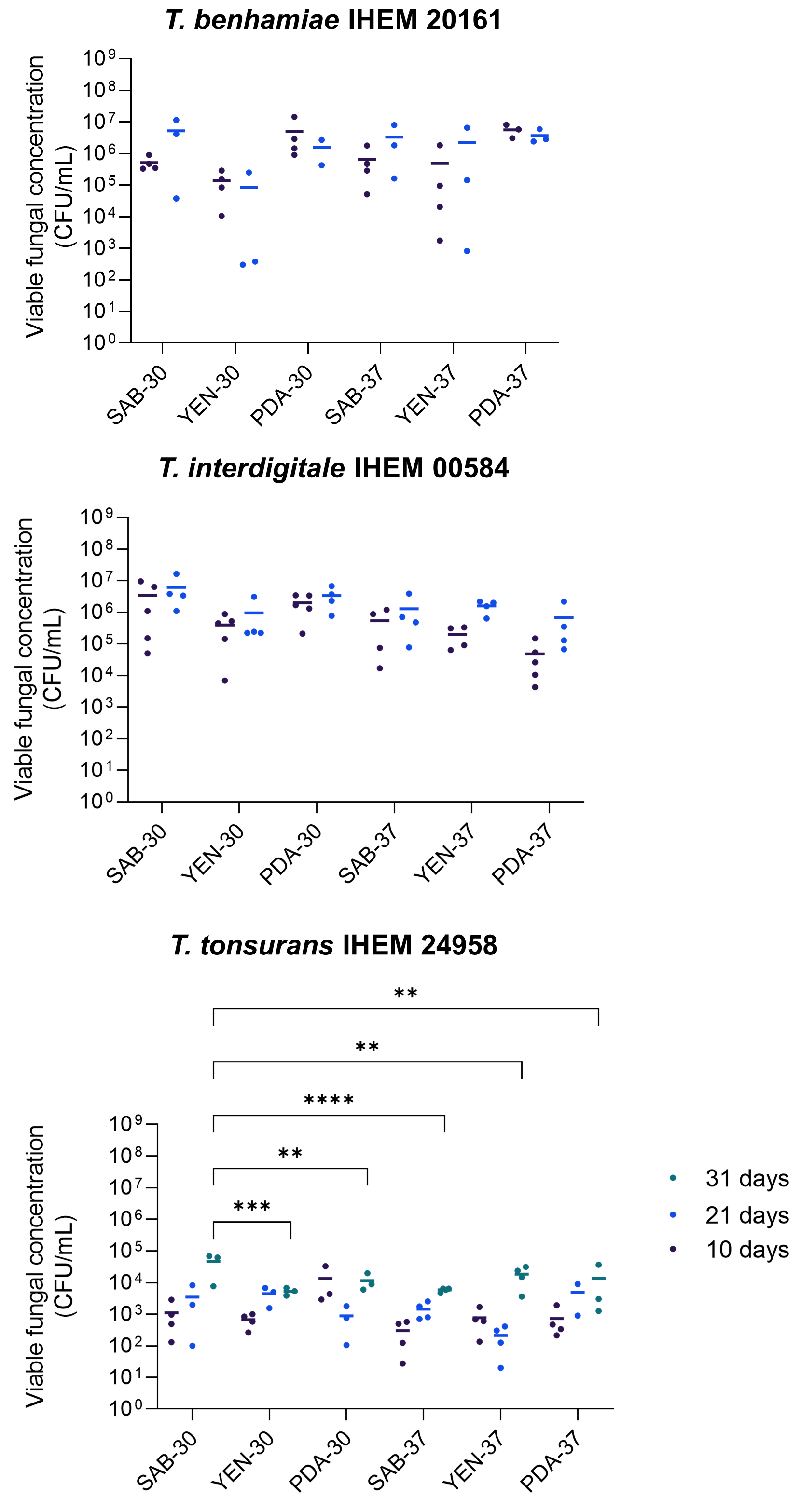

Supplement: Supplementary file 1 [file jof-07-01029-s001.zip › Faway, Staerck et al_Figure S5.tif]

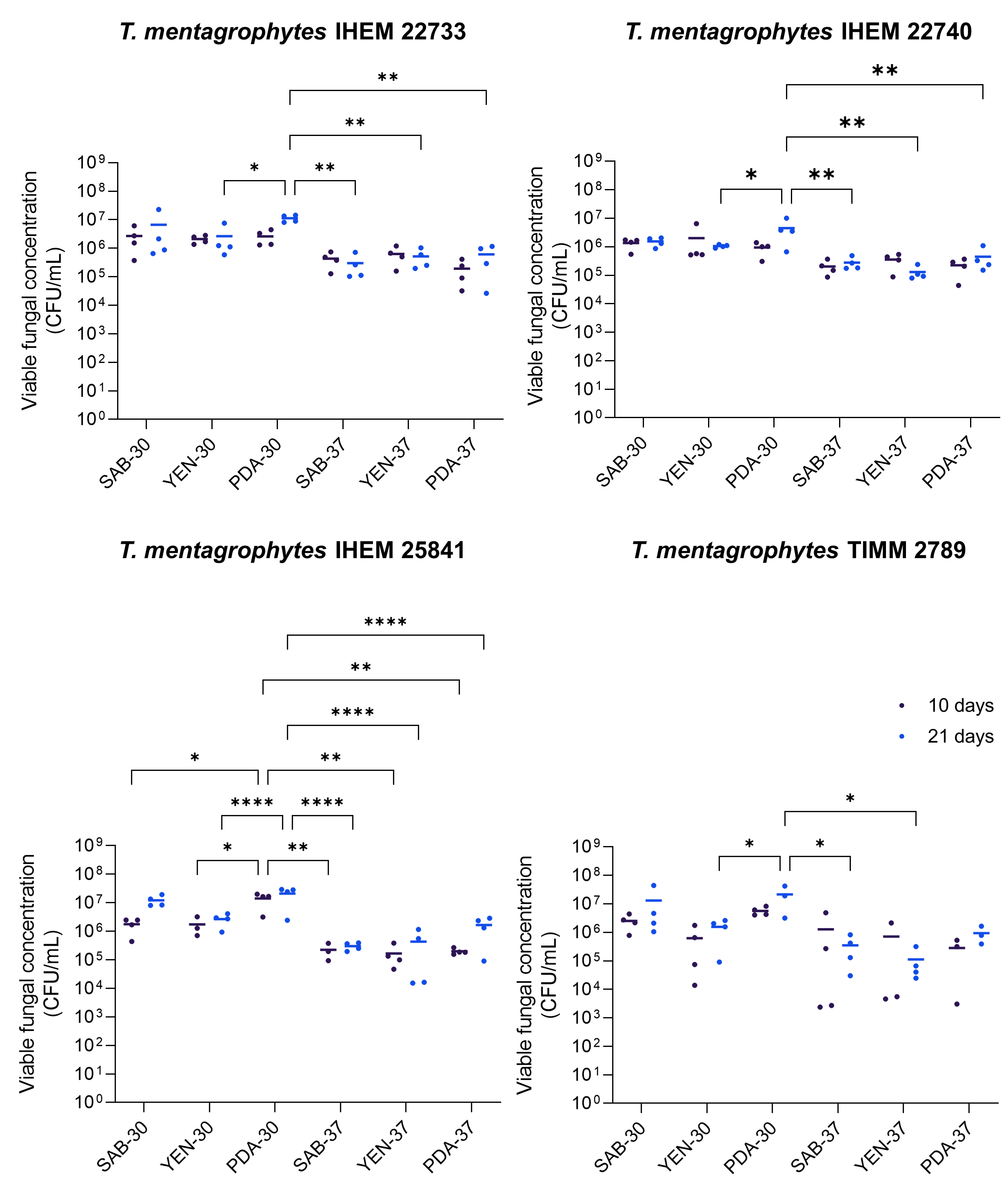

Supplement: Supplementary file 1 [file jof-07-01029-s001.zip › Faway, Staerck et al_Figure S6.tif]

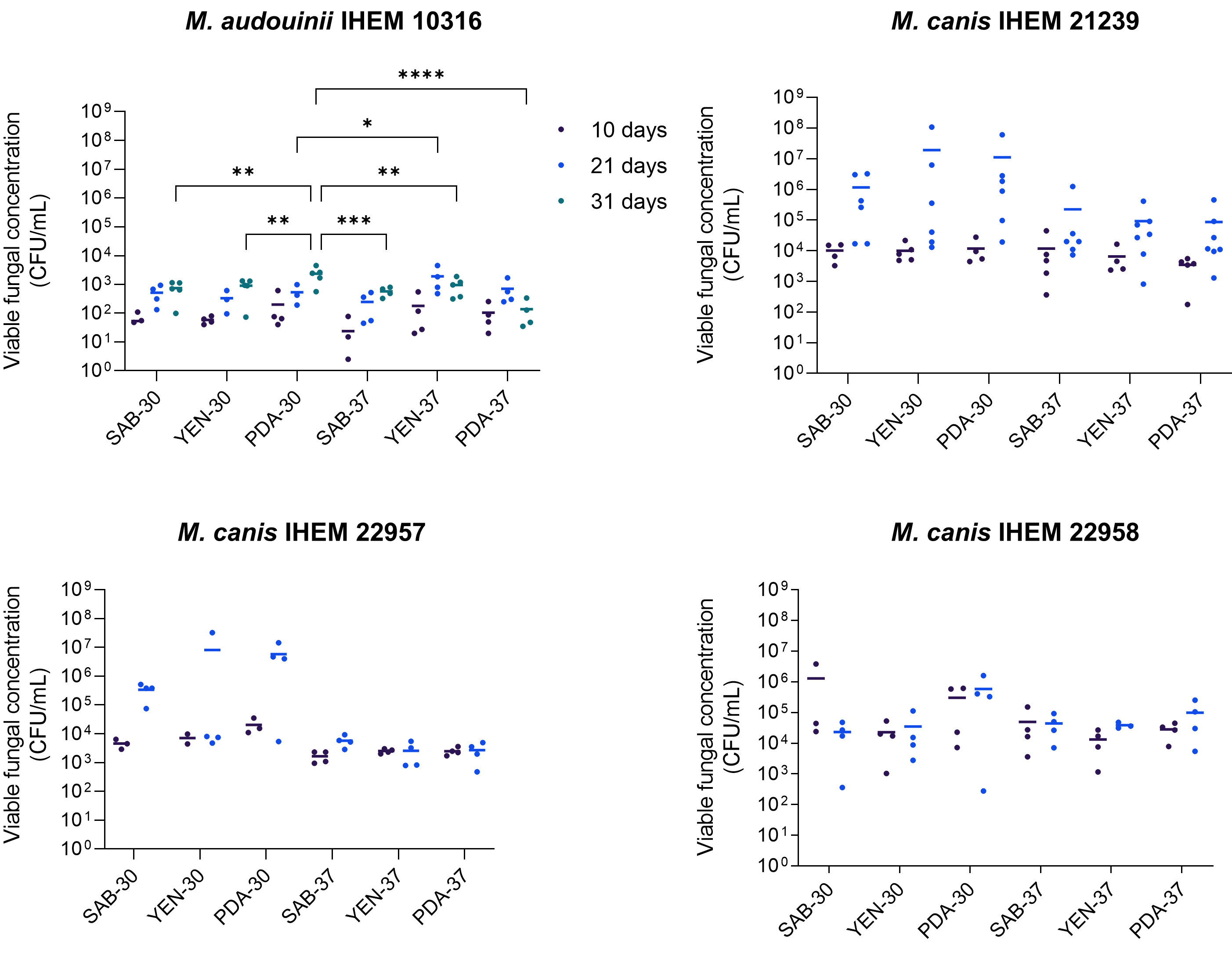

Supplement: Supplementary file 1 [file jof-07-01029-s001.zip › Faway, Staerck et al_Figure S7.tif]

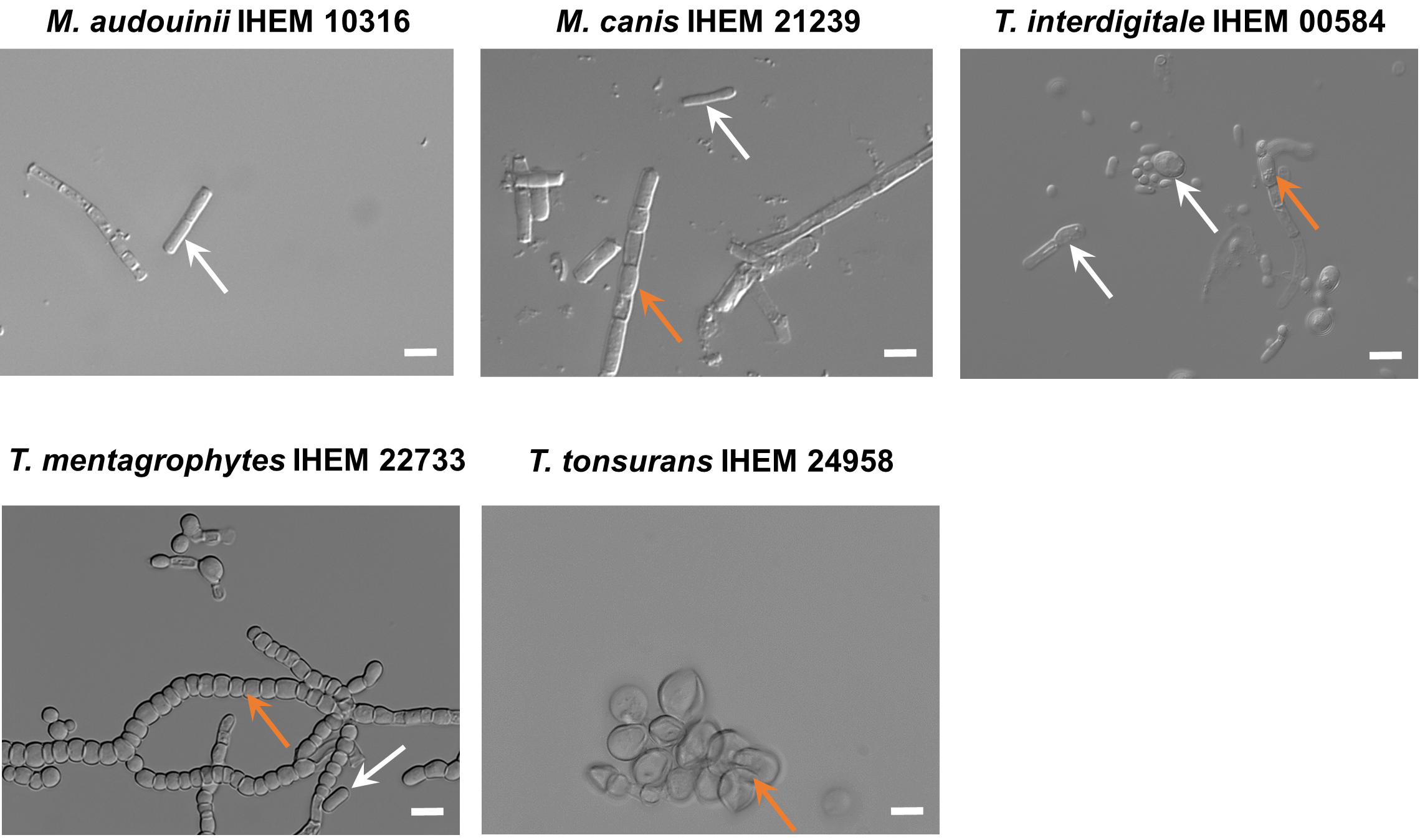

Supplement: Supplementary file 1 [file jof-07-01029-s001.zip › Faway, Staerck et al_Figure S8.tif]

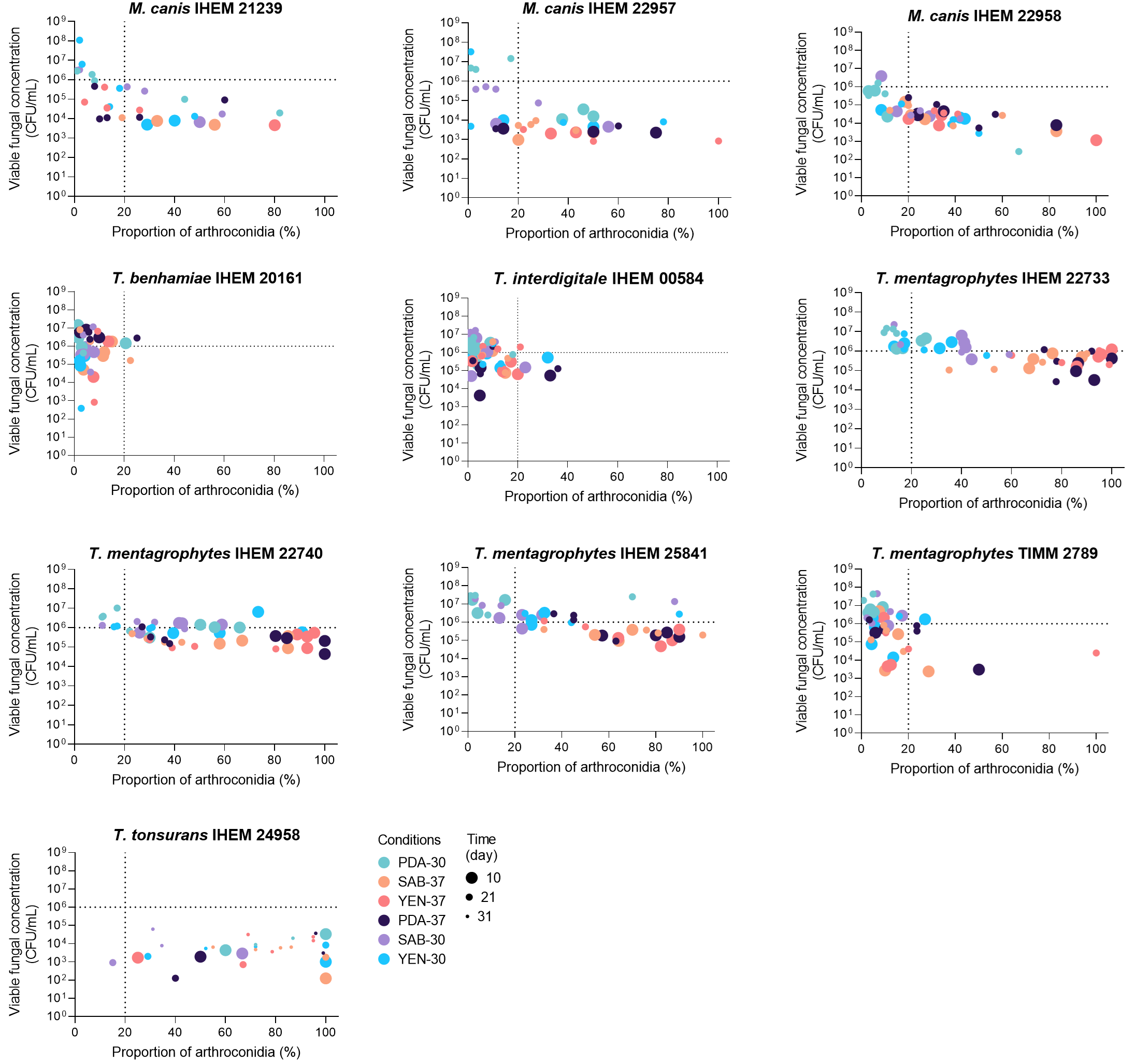

Supplement: Supplementary file 1 [file jof-07-01029-s001.zip › Faway, Staerck et al_Figure S9.tif]
